# Inference of *R*_0_ and Transmission Heterogeneity from the Size Distribution of Stuttering Chains

**DOI:** 10.1371/journal.pcbi.1002993

**Published:** 2013-05-02

**Authors:** Seth Blumberg, James O. Lloyd-Smith

**Affiliations:** 1 Fogarty International Center, National Institute of Health, Bethesda, Maryland, United States of America; 2 Department of Ecology and Evolutionary Biology, University of California, Los Angeles, California, United States of America; 3 F. I. Proctor Foundation, University of California, San Francisco, California, United States of America; Imperial College London, United Kingdom

## Abstract

For many infectious disease processes such as emerging zoonoses and vaccine-preventable diseases, 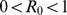 and infections occur as self-limited stuttering transmission chains. A mechanistic understanding of transmission is essential for characterizing the risk of emerging diseases and monitoring spatio-temporal dynamics. Thus methods for inferring 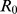 and the degree of heterogeneity in transmission from stuttering chain data have important applications in disease surveillance and management. Previous researchers have used chain size distributions to infer 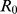, but estimation of the degree of individual-level variation in infectiousness (as quantified by the dispersion parameter, 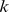) has typically required contact tracing data. Utilizing branching process theory along with a negative binomial offspring distribution, we demonstrate how maximum likelihood estimation can be applied to chain size data to infer both 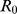 and the dispersion parameter that characterizes heterogeneity. While the maximum likelihood value for 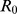 is a simple function of the average chain size, the associated confidence intervals are dependent on the inferred degree of transmission heterogeneity. As demonstrated for monkeypox data from the Democratic Republic of Congo, this impacts when a statistically significant change in 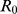 is detectable. In addition, by allowing for superspreading events, inference of 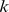 shifts the threshold above which a transmission chain should be considered anomalously large for a given value of 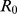 (thus reducing the probability of false alarms about pathogen adaptation). Our analysis of monkeypox also clarifies the various ways that imperfect observation can impact inference of transmission parameters, and highlights the need to quantitatively evaluate whether observation is likely to significantly bias results.

## Author Summary

This paper focuses on infectious diseases such as monkeypox, Nipah virus and avian influenza that transmit weakly from human to human. These pathogens cannot cause self-sustaining epidemics in the human population, but instead cause limited transmission chains that stutter to extinction. Such pathogens would go extinct if they were confined to humans, but they persist because of continual introduction from an external reservoir (such as animals, for the zoonotic diseases mentioned above). They are important to study because they pose a risk of emerging if they become more transmissible, or conversely to monitor the success of efforts to locally eliminate a pathogen by vaccination. A crucial challenge for these ‘stuttering’ pathogens is to quantify their transmissibility, in terms of the intensity and heterogeneity of disease transmission by infected individuals. In this paper, we use monkeypox as an example to show how these transmission properties can be estimated from commonly available data describing the size of observed stuttering chains. These results make it easier to monitor diseases that pose a risk of emerging (or re-emerging) as self-sustaining human pathogens, or to decide whether a seemingly large chain may signal a worrisome change in transmissibility.

## Introduction

There are many circumstances in infectious disease epidemiology where transmission among hosts occurs, but is too weak to support endemic or epidemic spread. In these instances, disease is introduced from an external source and subsequent secondary transmission is characterized by ‘stuttering chains’ of transmission which inevitably go extinct. This regime can be defined formally in terms of the basic reproductive number, 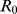, which describes the expected number of secondary cases caused by a typical infected individual. Stuttering chains occur when 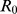 in the focal population is non-zero but less than the threshold value of one that enables sustained spread (i.e. 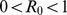). Transmission is therefore subcritical, and epidemics cannot occur. However there are many settings where such transmission dynamics are important. A major set of examples comes from stage III zoonoses, such as monkeypox virus, Nipah virus, and H5N1 avian influenza and H7N7 influenza [1]–[6]. Because most human diseases originate as zoonoses, there is significant public health motivation to monitor stage III zoonoses [7]–[10]. Subcritical transmission is also associated with the emergence of drug-resistant bacterial infections in some healthcare settings, such as hospital-acquired MRSA [11]. In addition, stuttering chains characterize the dynamics of infectious diseases that are on the brink of eradication, such as smallpox in the 1960s and 1970s [12] and polio now [13], [14]. Furthermore, stuttering chains are seen with measles and other vaccine preventable diseases when they are re-introduced to a region after local elimination [15]–[17].

A top priority in all of these settings is to quantify transmission, in order to determine the risk that the pathogen could emerge and become established in the human population of concern. This could occur due to demographic or biological changes that increase transmission, such as declining vaccine coverage [15] or pathogen adaptation [18]–[20]. Yet a recent review of transmission models for zoonotic infection identified a marked shortage of models that address the dynamics of zoonoses exhibiting stuttering chain transmission [4]. One major cause of this gap is that high-resolution data describing individual-level disease transmission are rare. The introduction events that trigger the stuttering chains are sporadic, and the transient nature of stuttering chains makes them difficult to track closely. Furthermore, contact tracing is logistically challenging because it requires rapid response surveillance teams and techniques for differentiating specific routes of disease transmission. In contrast, the total size of a transmission chain (i.e. the total number of cases infected) is much easier to obtain, since it does not require detailed contact tracing and can be assessed retrospectively based on case histories or serology. Accordingly, the most common data sets for stuttering pathogens are chain size distributions, which describe the number of cases arising from each of many separate introductions. Such data can be used to make estimates of 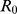 (or the ‘effective reproductive number’ in the presence of vaccination; for simplicity we will use the term 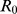 for all settings) [2], [15], [21]–[23]. This strategy has been applied successfully, particularly in the context of vaccine-preventable diseases, but one important simplification is that these analyses typically have not allowed for an unknown degree of heterogeneity in disease transmission among individual cases. This is an important omission, because individual variation in infectiousness is substantial for many infections [24] and can cause significant skews in the chain size distribution [25]. Thus it may be expected to affect conclusions about chain size distributions. For example, failure to account for superspreading events caused by highly infectious individuals can trigger false alarms in systems designed to detect anomalously large chains [2], [19].

We use simulations and epidemiological data to explore the influence of transmission heterogeneity on inference from chain size data, and to show that the degree of heterogeneity can actually be inferred from such data. Building upon prior studies we assume that the offspring distribution, which describes the number of secondary infections caused by each infected individual, can be represented by a negative binomial distribution. This has been shown to be an effective model for the transmission dynamics of emerging pathogens [24], and it encompasses earlier models (based on geometric or Poisson offspring distributions) as special cases. The negative binomial model has two parameters: the mean number of secondary infections, 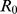, and the dispersion parameter, 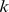, which varies inversely with the heterogeneity in infectiousness.

Knowledge of 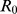 and 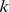 has important applications for stuttering chains, including quantifying the risk of endemic spread, predicting the frequency of larger chains, identifying risk factors for acquiring disease, and designing effective control measures. Such information helps to predict how changes in environmental or demographic factors might affect the risk of emergence. Meanwhile, the dispersion parameter alone is a useful measure of transmission heterogeneity, and serves as a stepping stone towards understanding whether heterogeneity arises from variance in social contacts, different intensities of pathogen shedding, variability in the duration of infectious period or some other mechanism.

Until now, estimation of individual variation in infectiousness (summarized by 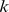) has depended on relatively complete contact tracing data, or on independent estimates of 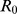 combined with the proportion of chains that consist of isolated cases [24]. While this approach has been successful, its application has been limited severely by data availability. Also it has sometimes led to internal inconsistencies within previous analyses, as for example when an 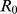 estimate predicated on the assumption that 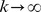 was used to obtain estimates of 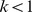 [24]. We show that maximum likelihood (ML) approaches can be used to estimate 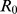 and determine reliable confidence intervals from stuttering chain data, while allowing for an unknown amount of heterogeneity in transmission. The relationship between 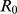, 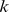 and the chain size distribution has been derived for varying degrees of heterogeneity [22], [23], but none of these studies has treated 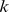 as a free parameter and this introduces a wildcard into the inference process. By providing a unified framework for inference of 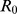 and 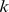, we prevent such difficulties.

We demonstrate the epidemiological significance of our ML approach by analyzing chain size data obtained during monkeypox surveillance in the Democratic Republic of Congo from 1980–1984 [26], [27]. Monkeypox is an important case study for these methods, because recent reports indicate that its incidence has increased 20-fold since the eradication of smallpox in the late 1970s [28], raising the urgent question of whether the virus has become more transmissible among humans. Meanwhile, challenging logistics make the collection of follow-up data difficult and resource-intensive. Fortunately, surveillance data from the 1980s data set is unique in its detail and it allows us to demonstrate how chain size data yields results that are consistent with harder-to-obtain contact tracing data. This suggests that future monitoring of 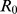 can be achieved by monitoring chain size data by itself. We demonstrate that accurate knowledge of the dispersion parameter is important for reliably determining when an apparent change in transmissibility is statistically significant. In addition, our focus on chain size distributions permits us to determine quantitative thresholds for chain sizes that can be used during surveillance to decide if a particular transmission chain is unusually large and likely to indicate an abrupt increase in 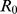. Such indications can facilitate targeted, cost-effective implementation of control measures. Lastly, we consider the real-world difficulties that can arise in obtaining transmission chain data, including the possibility that cases remain unobserved and the complications of overlapping transmission chains. We present a summary of when such observation errors can interfere significantly with reliable inference of transmission parameters.

## Results/Discussion

We define a ‘stuttering transmission chain’ as a group of cases connected by an unbroken series of transmission events. Transmission chains always start with a ‘spillover’ event in which a primary case (sometimes referred to as an index case) has been infected from an infection reservoir outside the population of interest. Mechanisms of spillover differ among pathogens and circumstances, but include animal-to-human transmission, infection from environmental sources or geographical movement of infected hosts. The primary case can then lead to a series of secondary cases via human-to-human transmission within the focal population. Sometimes no secondary transmission occurs, in which case a transmission chain consists of a single primary case. We define an infection cluster as a group of cases occurring in close spatio-temporal proximity, which may include more than one primary infection and thus be composed of more than one transmission chain. Some authors use ‘outbreak’ or ‘infection cluster’ for what we call a transmission chain.

### Comparison of contact tracing and chain size analysis

To characterize the transmission of subcritical diseases, epidemiologists might record data describing the total disease incidence, the number of cases in each transmission chain, the number of transmission generations in each transmission chain, or complete contact tracing data. Because the collection of high-resolution epidemiological data is resource and labor intensive, there is great benefit to understanding the type and quantity of data needed for a specific type of assessment. For instance, total incidence data on its own is not sufficient to infer human-to-human transmissibility for subcritical infections, because the contribution of spillover cases is unspecified. However, chain size and contact tracing data can be used to infer 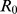. In fact, for our negative binomial model of disease transmission, the ML estimate of 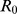 is identical when the likelihood is based on either chain size data only, chain size data coupled with knowledge of the transmission generation when the chain went extinct, or complete contact tracing data (see methods). This shows that for the purpose of estimating 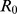, chain size data can be equivalent to contact tracing data. However these theoretical observations must be placed in proper context as contact tracing is often valuable for many other reasons, such as helping to ensure data quality and minimizing unobserved cases.

**Figure 1:**
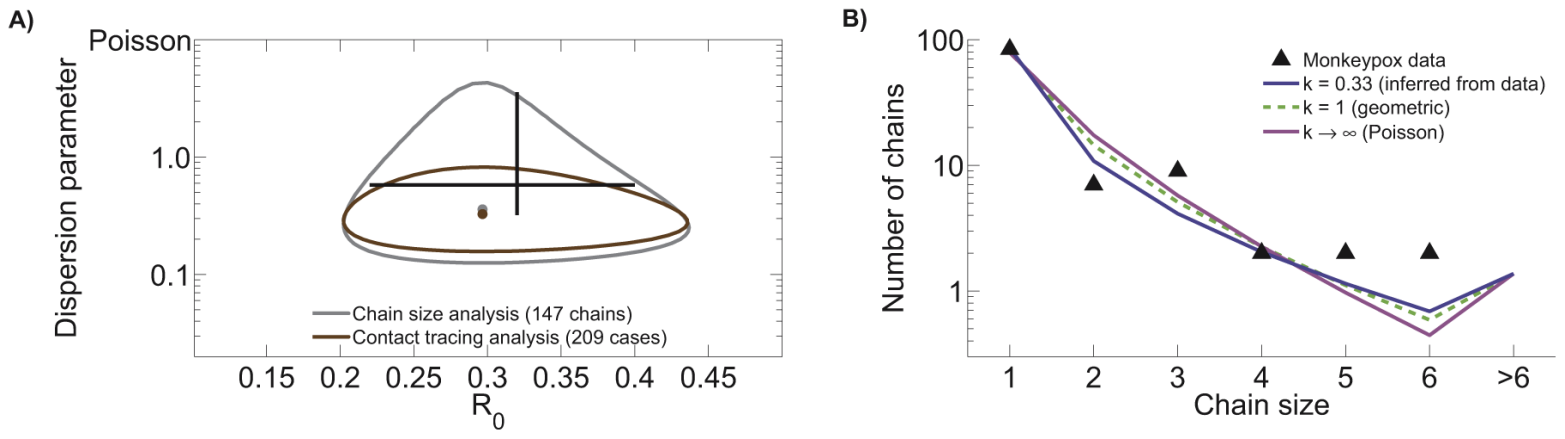
Contact tracing and chain size analysis of monkeypox data. A) Ninety percent confidence regions for 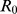 and 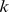 inference are shown for monkeypox data gathered between 1980 and 1984 in the Democratic Republic of Congo [26], [27]. The two confidence regions are based on the same set of data. The chain size analysis is based on the number of cases in isolated outbreaks of monkeypox, whereas the contact tracing data are based on the number of transmission events caused by each case. The black cross hairs indicate the previously reported 90% confidence intervals for monkeypox transmission parameters based on the first generation of transmission in this data set [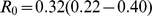, 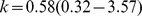] [24]. B) Model predictions for the chain size distribution based on three different values of 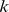, including the ML value of 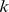 that is based on contact tracing data. A subset of the chain size data consisting of only those chains having exactly one identified primary infection is shown for comparison to model predictions. When the two-parameter ML value for the contact tracing data is compared to the likelihoods of the 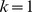 and 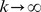 models, the 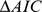 scores for the latter models are 4.3 and 23.3 respectively.

The detailed and accurate data describing human transmission of monkeypox virus in the 1980s [26], [27] provide an opportunity to compare the result obtained by inferring 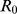 and 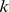 from chain size data to those obtained from contact tracing data. Inference results show that the confidence region obtained from contact tracing data is nested within that obtained from chain size analysis (figure 1A and table 1). In fact, the ML value for 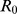 and the associated univariate confidence intervals are identical for the two methods. Meanwhile, the ML value for 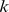 is similar for the two methods, but the confidence interval is broader for chain size analysis than for contact tracing analysis. When compared to previous inference results [24] our chain size and contact tracing estimates for 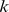 tend to lower values (though confidence intervals overlap). Since the previous results were based entirely on the first generation of transmission, this indicates that transmission of secondary cases may be more variable than transmission by primary cases.

**Table 1:** Inference results for monkeypox data.

	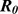	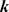
ML value for chain size analysis	0.30	0.36
90% CI for chain size analysis	0.22–0.40	0.16–1.47
95% CI for chain size analysis	0.21–0.42	0.14–2.57
ML value for contact tracing analysis	0.30	0.33
90% CI for contact tracing analysis	0.22–0.40	0.19–0.64
95% CI for contact tracing analysis	0.21–0.42	0.17–0.75

The chain size distribution predicted by models fitted under various assumptions about transmission heterogeneity exhibit subtle, but important differences (figure 1B). Overall, allowing a flexible amount of transmission heterogeneity produces a model that has a higher proportion of isolated cases and larger chains, but a lower proportion of intermediate-sized chains. Meanwhile, all of the models are compatible with a higher proportion of longer chains (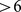 cases) than were actually observed. This suggests that household structure or some other factor may act to reduce transmission after chains reach a moderate size (possibly because the local pool of susceptibles is depleted), but the data do not support a definitive conclusion.

### Monitoring change in 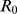 can be accomplished with chain size data

**Figure 2:**
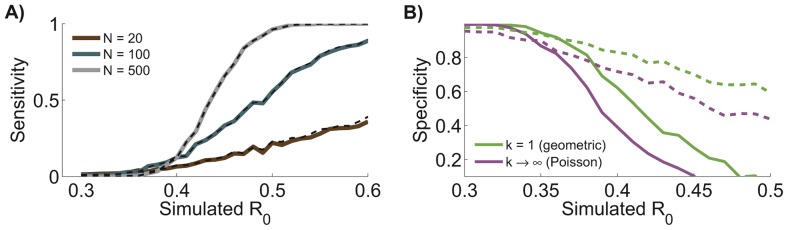
Chain size analysis clarifies surveillance needs. A) Applying maximum likelihood estimation to simulated data shows the sensitivity of chain size analysis and contact tracing analysis for detecting a change in 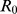. Results show the probability of detecting a significant change between the monkeypox data from the 1980s and a simulated data set with 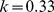 (equal to the ML value for the 1980s data) and 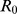 specified along the x-axis. Statistical significance was determined by setting a 95% confidence threshold on the likelihood ratio test (details provided in methods section). Curves represent different values for the number of simulated chains, 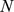. Results are depicted for inference from detailed contact tracing data (dashed line) or more readily available chain size data (solid line). B) The specificity for detecting a statistically significant change in 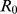 (as compared to 1980s monkeypox contact tracing data) is shown when various values of 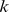 are assumed during chain size analysis (as applied to the same chain size data simulated for panel A). The specificity is defined as the probability that a change is not detected for an assumed value of 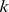 conditioned on our gold standard for a lack of change (e.g. a change is not detected when 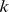 is allowed to be a free parameter during inference). The solid line corresponds to 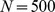 chains and the dashed line to 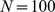.

When incidence of an emerging disease increases, a frequent goal of surveillance is to assess whether this is attributable to a rise in transmissibility in the focal population, as manifested by an increased 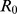. For instance, the observed 20-fold rise in incidence of human monkeypox [28] might be explained by an increased 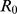 in the human population or by an increase in animal-to-human transmission. Since a relatively low incidence limits the data available for monkeypox (and many other subcritical diseases), it is helpful to determine how the type and quantity of data impacts the ability to detect a specific change in 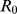. Utilizing the results of 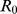 and 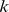 inference for monkeypox in the 1980s, we can ascertain how the power to detect a statistically significant change in 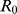 varies with the size of the data set and the magnitude of the change in 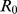 (figure 2A). As expected, the more data that are available, the more statistical power there is to detect a change in 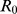. The sensitivity of chain size analysis for detecting a change in 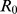 is almost identical to that of contact tracing analysis (when allowing 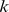 to be a free parameter in both analyses). This suggests that when faced with a trade-off, monitoring of 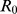 is enhanced more by obtaining additional data on chain sizes (provided the sizes are accurately assessed) than by obtaining detailed contact tracing on a subset of available data.

Equally as important as detecting a change in 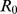 is knowing when there may be an inaccurate report of a change. In the case of monkeypox, we find that assuming an incorrect level of transmission heterogeneity in a chain size analysis can lead to over-confident detection of a change in 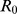 relative to the 1980s data. This is because under-estimating the degree of transmission heterogeneity leads to inappropriately narrow confidence intervals for the estimated 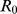. Over-confident detection of a change in 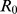 is most worrisome when two data sets simulated using identical parameters give rise to distinct estimates of 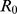 more often than expected (table 2). This over-confidence arising from incorrect assumptions about 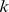 can also lead to a lack of specificity for detecting a change in 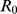 in simulated data sets, when inference based on letting 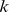 be a free parameter is used as the gold standard (figure 2B). While it could initially appear preferable that incorrect 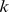 values can lead to greater probabilities of detecting changes in 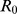, this trades off against the higher rate of false positive detections and a general loss of statistical integrity (e.g. the coverage of confidence intervals will not match the nominal levels).

**Table 2:** Probability of falsely detecting a change in 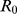.

Number of chains simulated	Percentage when 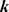 inferred	Percentage when 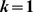	Percentage when 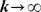
20	1.7	10.2	14.9
100	5.0	10.8	15.5
500	5.1	10.8	15.7

As detailed in the methods section, a statistical difference was determined by using the likelihood ratio test to compare two transmission models. The first model assigns separate values of 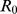 to the 1980s monkeypox data and the simulated data, while the second model assigns a single 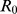 to both data sets. Both models assign a single value of 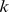 to both data sets. Since the second model is nested in the first, statistical significance was determined by setting a 95% confidence threshold on the likelihood ratio test. Probability values that exceed 5% indicate an over-abundance of false positive detections of change in 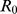. Each result was based on 10,000 simulations.

### Chain size thresholds provide an alternative approach to detecting change in 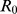


**Figure 3:**
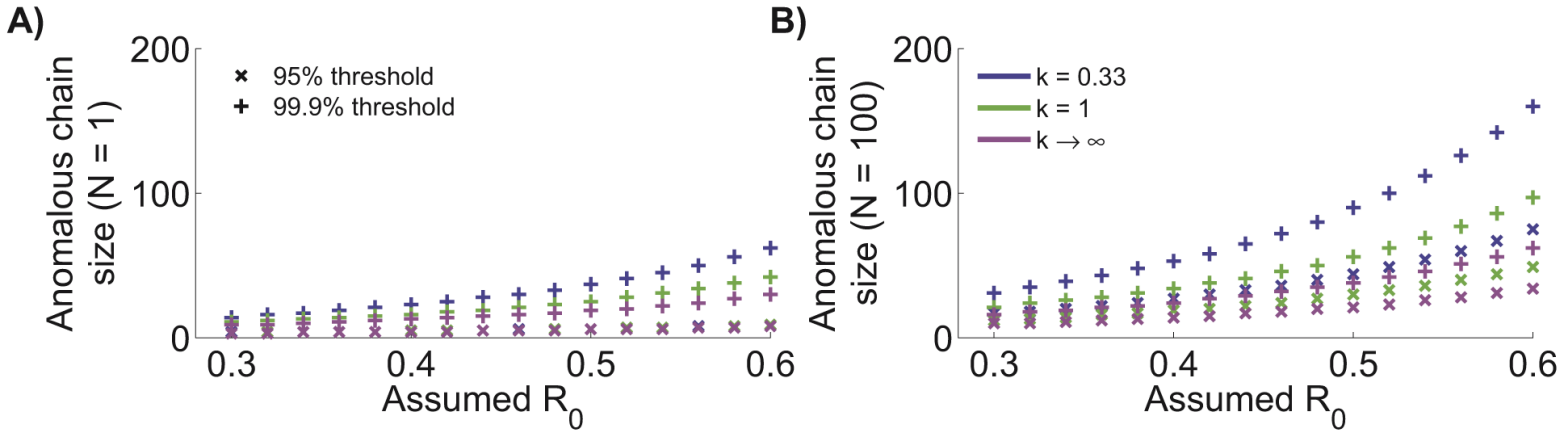
Size of anomalously large chains. A) Size of an observed chain that is anomalously large as a function of 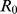 and 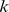. The cumulative distribution threshold indicated in the legend denotes the chosen cutoff for the cumulative chain size distribution that determines when an observed chain is anomalously large. B) Analogous to panel A, but results are based on the largest observed chain for a group of 100 observed chains.

For many surveillance systems, large chains are more likely to be detected than isolated cases. This could give rise to biases in the chain size distribution data, which we address in a later section. In these situations, an alternative approach to detecting a change in 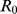 is to determine the size of the largest chain that would be expected by chance (for some arbitrary threshold in the cumulative probability distribution) [2]. The size cutoff for what is then considered an anomalously large chain depends on the values of both 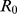 and 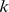 (figure 3). As the assumed value of 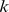 decreases, the chain size that is considered anomalously large will rise because superspreading events become more frequent. If chain size probabilities are calculated using traditional assumptions of 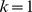 or 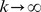, then too many false alarms may be raised concerning the number of chains that are perceived to be anomalously large, particularly for pathogens that exhibit significant transmission heterogeneity. The determination of a chain size cutoff also depends on whether the detection of large chains is based on individual reports versus the investigation of the largest chains in a collection of surveillance data (compare figures 3A and 3B).

In some situations, a rapid response protocol might be instituted to quickly investigate worrisomely large chains. In this case, an anomalous size cutoff can be chosen based on there being real-time reports of the size of single chains (as distinct from considering the largest chain obtained from an entire surveillance data set). However, assuming an incorrect value of 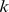 could trigger many false alarms for chains that are actually consistent with known transmission patterns (table 3). For instance if we assume that monkeypox transmission follows the parameters estimated with our ML model (blue line of figure 1B), then for a 99.9% cumulative distribution threshold setting 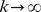 will result in five-fold more chain investigations than if 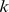 is set at the ML value of 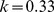.

**Table 3:** Frequency of anomalously large chains.

Cumulative distribution threshold	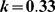 assumed	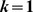 assumed	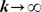 assumed
95%	4.9% ( 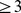 cases)	4.9% ( 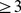 cases)	4.9% ( 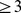 cases)
99%	0.88% ( 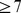 cases)	1.29% ( 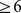 cases)	1.94% ( 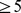 cases)
99.9%	0.09% ( 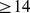 cases)	0.22% ( 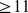 cases)	0.43% ( 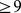 cases)

The cutoff for a chain sizes that are considered anomalously large was determined by when the cumulative chain size probability exceed the cumulative distribution threshold for 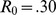 (ML value for 1980s monkeypox data) and 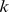 as indicated in the table. The frequency of outlier detection was then determined according to the probability that chain sizes would exceed the chain size cutoff as predicted by the ML values of 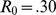 and 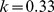 for monkeypox.

In other situations, chain sizes may be evaluated collectively after a predefined period of surveillance. For the ML values of 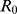 and 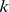 estimated for monkeypox in the 1980s, the cumulative distribution of chain sizes shows that there is a 95% chance that the largest of 100 observed chains will be less than 17 cases and a 99.9% chance that the chains will all be less than 31 cases. These results suggest cutoffs for chain sizes that deserve increased investigation (17 cases) and provides a chain size cutoff for determining when 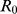 has almost certainly increased (31 cases). This contrasts with the 95% and 99.9% chain size cutoffs of 10 and 16 obtained when 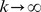 is assumed.

### Characterizing maximum likelihood inference of 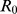 and 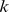 from chain size distributions

By demonstrating the concordance of results based on chain size and contract tracing data when inferring 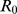 and 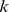, our analysis of monkeypox data provides motivation to further characterize the performance of inference based on chain size data. To evaluate the accuracy and precision of ML inference of 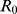 and 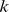 from chain size data, we ran simulations for various combinations of 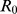, 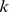, and number of observed transmission chains, 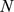. For each simulated dataset, we determined the ML 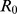 and 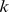 values (equations 9, 11 and 12) and evaluated whether the realized coverage probability of the 90% confidence intervals conformed to expectations (equations 22).

**Figure 4:**
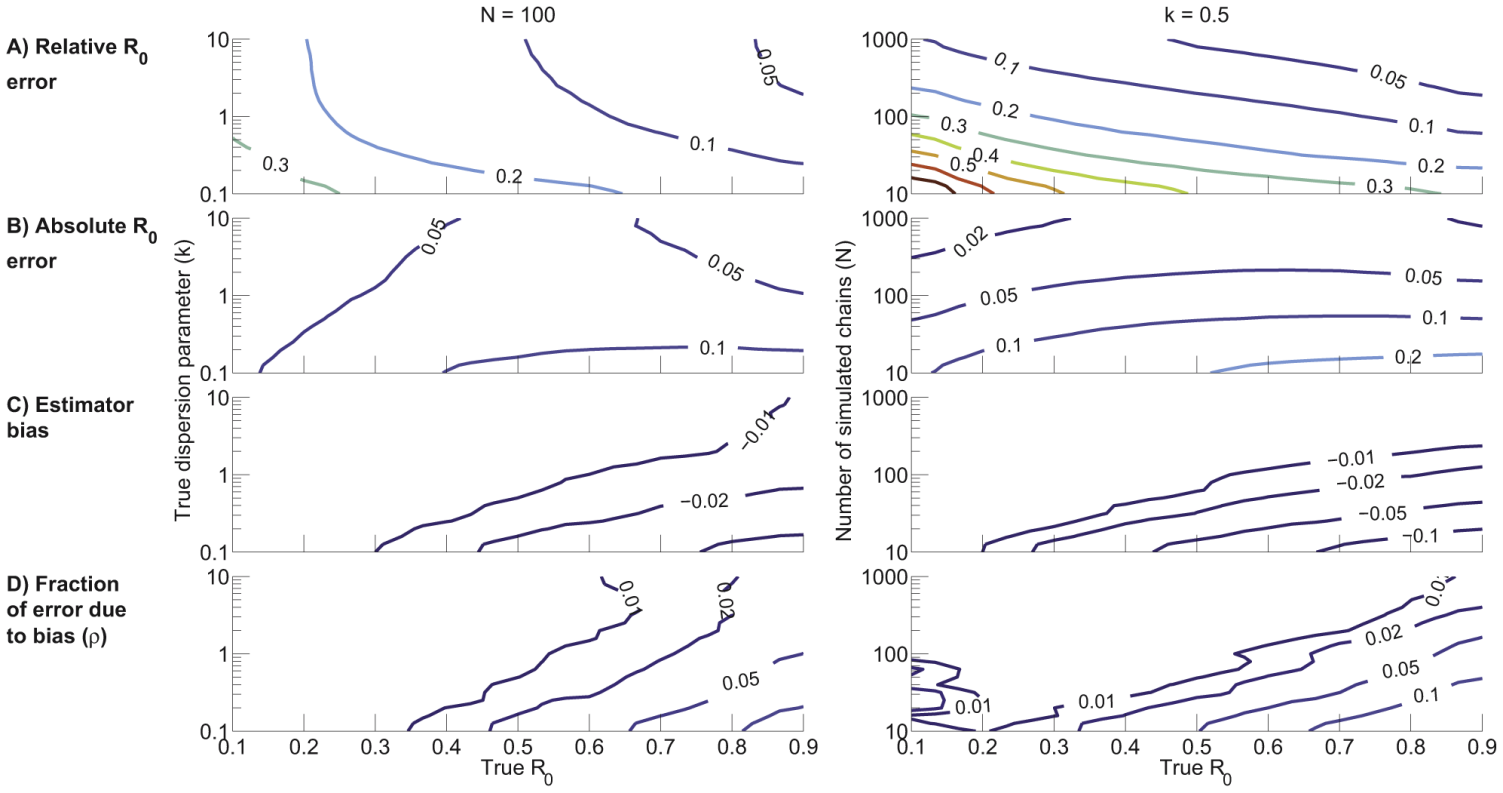
Characterization of 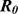 inference as a function of 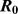 and 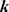 with 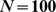 (left column) and as a function of 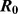 and 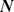 with 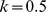 (right column). The axes represent the true 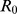, 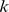 and 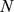 inputs for the simulations. A) Root mean square relative error for ML inference of 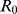 (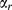). B) Root mean square absolute error for ML inference of 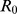 (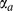). C) Bias of 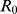 inference (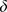). D) Fraction of the 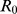 absolute error that is attributable to bias (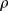). The contour plots were generated based on a lattice of simulation results for linearly spaced values of 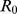 and logarithmically spaced values of 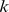 or 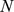. The values for each lattice point were computed by averaging the results of 2,000 simulations. For visualization purposes, simulation results were smoothed by a one-neighbor moving average.

Due to the challenges of illustrating the dependence of inference error on three variables, this section considers two special cases of parameter values. First we fix 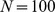 and consider how the inference error depends on 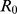 and 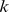 (figure 4 - left column). This provides an assessment of error bounds when a realistic amount of data is available and when there is no prior information on 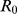 or 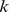. Next we fix 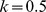 and consider how the inference error depends on 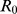 and 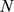 (figure 4 - right column). This scenario highlights the relationship between inference accuracy and data set size when a significant amount of transmission heterogeneity is present. Qualitatively similar results are obtained when fixing different values for 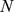 or 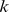 (data not shown).

We limit our simulation results to 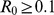 because when 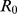 is close to zero there are too few secondary infections for inference to be meaningful. We also limit ourselves to 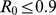 because large stuttering chain sizes become increasingly likely when 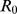 approaches one, and so our modeling assumption that transmission is independent of stuttering chain size becomes increasingly dubious. Consistent with the range of inferred 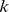 from prior analysis of a variety of infectious diseases, we restrict our analysis to 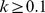 [24]. Meanwhile, we focus on 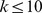 since 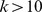 is similar to the Poisson distribution limit of 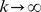 [29]. Lastly, we choose a range of 10 to 1000 for 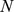 since this reflects the size of most empirical data sets.

#### Inference of 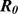 from chain size distributions exhibits little bias

We summarized the error in 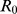 inference using the root mean square of the relative and absolute errors, 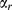 and 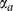 (equations 14 and 15). The relative error 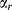 increases as 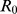 decreases, owing to the smaller denominator, and 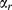 also increases as 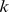 decreases because of increased variation arising from stochasticity as the offspring distribution becomes more skewed (figure 4A - left column). Meanwhile since ML inference is asymptotically unbiased, 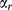 decreases as the data set size increases (figure 4A - right column).

As with relative error, the absolute error 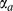 increases as 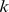 decreases. In contrast to 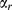, the dependence of 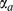 on 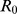 is relatively weak for high values of 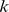 and 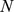 (figure 4B). However, if significant heterogeneity is present or when the data set is small, then 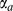 grows as 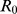 increases. As with relative error, 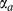 tends to zero for large data sets.

To further our understanding of the error in 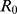 inference, we computed the bias and standard deviation arising in ML inference of 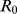. The former is a measure of accuracy and is potentially correctable, while the latter is representative of imprecision inherent in stochastic processes and is uncorrectable. The bias of ML inference of 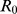 (figure 4C) is due to discrepancy between the observed and predicted average chain size. The bias is always negative due to the non-linearity of equation 12, which makes 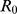 inference more sensitive to underestimates of the average chain size than to overestimates. The amplification of bias seen with decreasing 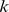 arises because greater transmission heterogeneity tends to produce chains that are either very small or very large, thus accentuating the influence of Jensen's inequality on equation 12. Similarly, the magnitude of the bias increases for small 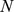 because the stochastic nature of small data sets results in a larger sampling variance of the observed average chain size.

In principle, bias-correction could be applied to 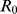 inference. However, this would be hard to do in a self-consistent manner because the bias depends on 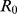. To decide whether the extra effort is worthwhile, it is instructive to know the fraction 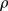 by which 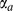 would decrease if bias were eliminated (equation 18). This fraction increases as 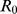 increases, 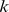 decreases, or 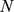 decreases (figure 4D). However 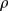 remains less than 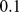 for a large region of parameter space. Therefore, given other uncertainties in data acquisition and analysis, it seems that bias correction will rarely be worthwhile.

#### Transmission heterogeneity can also be reliably inferred from chain size distributions

**Figure 5:**
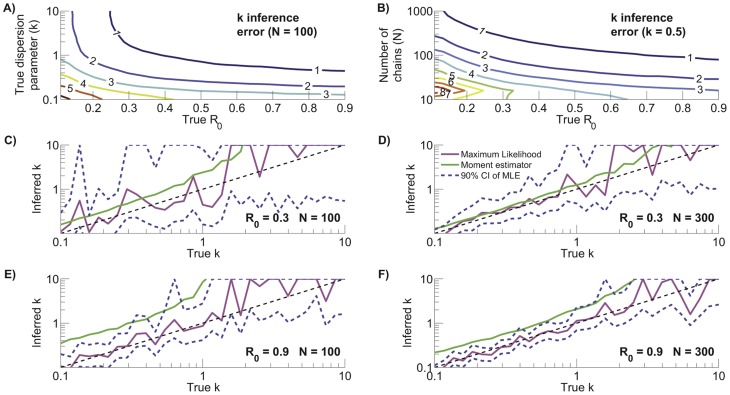
Characterization of 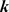 inference. A) Error of 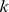 inference as quantified by the root mean square of the absolute differences between the reciprocals of the inferred and true value of 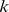 for simulated data (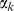). The contour plot was generated based on the same simulations and inference procedure that was used to produce the 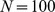 panels of figure 4. B) Same as panel A except that 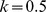 for all simulations and now the number of simulated chains varies. C) Summary of how well 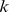 inference works when 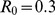 and 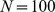. The dashed black line represents a perfect match between the true and inferred 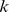 values. The magenta line shows the median value of ML inference of 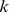. The dashed blue lines show the median values of the upper and lower limits of the 90% confidence intervals for 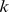. For visualization purposes and because 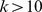 is essentially a Poisson distribution, the upper confidence intervals were bounded at 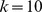. The green line shows the median estimate for 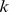 inference based solely on the first and second moments of the simulated data, 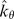 (equation 7). All curves were determined from the results of one thousand simulations for logarithmically distributed values of the true 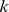. D–F) Same as panel C, but for different 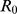 and 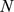 pairs.

Assessing inference of transmission heterogeneity is complicated by the inverse relationship between 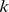 and the variance of the offspring distribution. Thus we measure the error of 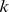 estimation in relation to 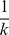 (equation 16). This emulates earlier work on ML estimation of 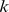, both as a general biostatistical problem and from contact tracing data [29], [30]. In broad terms, the error of estimating 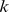 from chain size data (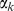) decreases as 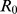 and 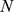 increase (figures 5A and 5B). This is explained by there being more observed transmission events that provide information on transmission patterns. Meanwhile, the error tends to increase with decreasing 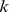. This is likely due to a need for relatively large sample sizes to observe the rare superspreading events that are characteristic of low 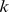 values [29]. Some caution is needed in interpreting this trend because our error metric of 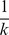 increases as heterogeneity increases. However it is unlikely that this trend is an artifact of our chosen metric because it is also seen when other error metrics are used, such as the difference between the inferred and true coefficient of variation (data not shown).

Because of the non-intuitive relationship between 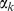 and confidence intervals for 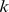, we have illustrated the performance of 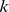 inference for four specific choices of 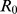 and 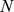 (figures 5C–5F). These plots reinforce the trends seen in panels A and B. In particular, narrower and more consistent confidence intervals for large 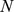 and large 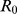 support the conclusion that this region of parameter space allows the most precise and accurate inference of 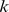. The confidence intervals are also narrower for smaller 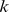. However, this does not accurately reflect the uncertainty in the degree of transmission heterogeneity because small changes in small values of 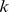 can significantly change the offspring distribution's coefficient of variation. In contrast, the more rugged curves for ML inference when 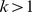 should be interpreted with consideration of the offspring distribution changing minimally for higher values of 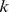. Despite the inherent difficulties of inferring low values of 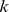, the ML approach appears robust because there is no discernible bias of the ML estimate of 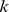 and the median confidence intervals consistently include the true values of 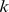.

Motivated by the observation that the ML estimator for 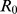 is a simple function of the average chain size, we explored whether accurate inference for 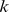 can be obtained by considering just the first two moments of the chain size distribution (equation 7, figures 5C–5F). Second moment inference improves as 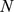 increases, but there is a clear bias towards over-estimation of 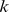. The non-negligible bias suggests that whenever possible it is preferable to estimate 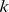 by ML inference from the full distribution of chain sizes.

#### Confidence intervals show accurate coverage

Since confidence interval calculations are independent of the particular metric used for quantifying inference error (e.g. insensitive to our use of 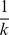 for our error metric), their coverage accuracy provides a useful assessment of ML inference [31]. For most of the 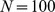 and 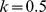 parameter space slices, the 90% coverage probability of 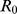 estimates varied from 88% to 93% and tended to increase with increasing 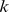 (data not shown). This coverage probability is consistent with the expected value of 90%. The one exception was for 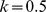, 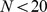 and 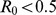 when the coverage probability rose as high as 98%. This occurred because confidence intervals got wider for these small data sets, and not because 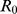 inference was more precise. The coverage probabilities for confidence intervals of 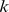 estimates show similar concordance. As with the 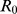 estimates, when 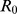 and 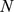 are both low, the coverage probability for 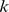 tended to be higher than the nominal level of 90%. It was also too high when 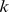 approached higher values, but this is likely due to the boundary effects when 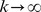. The take-home message is that for most of parameter space, the confidence intervals for 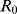 and 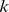 inference can be trusted when ML inference is applied to high quality data.

Overall, our characterization of the inference of 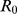 and 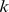 from the size distribution of stuttering chains shows that estimation accuracy is more likely to be limited by data or shortcomings of our modeling assumptions than by biased inference. For simulated data over a wide range of parameter values, inference of 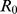 has an error of less than 10%, negligible bias and reliable confidence intervals. Inference of 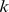 also has reliable confidence intervals, but unlike 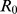, the parameter itself is typically not the direct focus of epidemiological interest. Thus caution is needed in interpreting the absolute error in 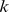 estimates, due to the nonlinear relationship between 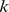 and the coefficient of variation and other measures of heterogeneity for the offspring distribution.

### Data limitations have variable impact on inference results

The preceding analyses have shown the potential for accurate inference of transmission parameters from chain size data, but we have not yet considered how imperfect case detection impacts inference results. We have also ignored complications arising when multiple chains are mixed into a single cluster. This latter scenario allows the possibility that some primary infections are falsely classified as secondary cases. Here we consider whether and how these types of data limitations impact inference results.

#### The bias arising from imperfect observation depends on which cases are unobserved

No surveillance system is perfect and some cases will be missed. However the mechanisms underlying imperfect observation can alter 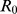 estimation in different ways [2]. For instance, if the observation of each case is independent of all other cases, then the average observed size of a chain will be smaller and the resulting 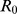 estimates will be smaller. However, other processes such as retrospective investigation can paradoxically increase the average observed chain size and thus lead to higher estimates of 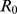.

By modeling observation as a two-step process, we can explore the impact of a diverse range of scenarios. We define the passive observation probability as the probability that any case will be detected by routine surveillance measures. This probability applies independently to all cases, so multiple cases in the same chain can be detected by passive surveillance. In some settings, there is an active surveillance program that investigates outbreaks that have been detected by the passive system. We define the active observation probability as the probability that a case will be detected by active surveillance, conditional on that case not having been detected by passive surveillance. Cases can be detected by active surveillance only if they belong to a transmission chain where at least one case is detected by passive surveillance. (When the active observation probability is zero or one, respectively, our observation model maps onto the ‘random ascertainment’ and ‘random ascertainment with retrospective identification’ scenarios previously analyzed [2].).

**Figure 6:**
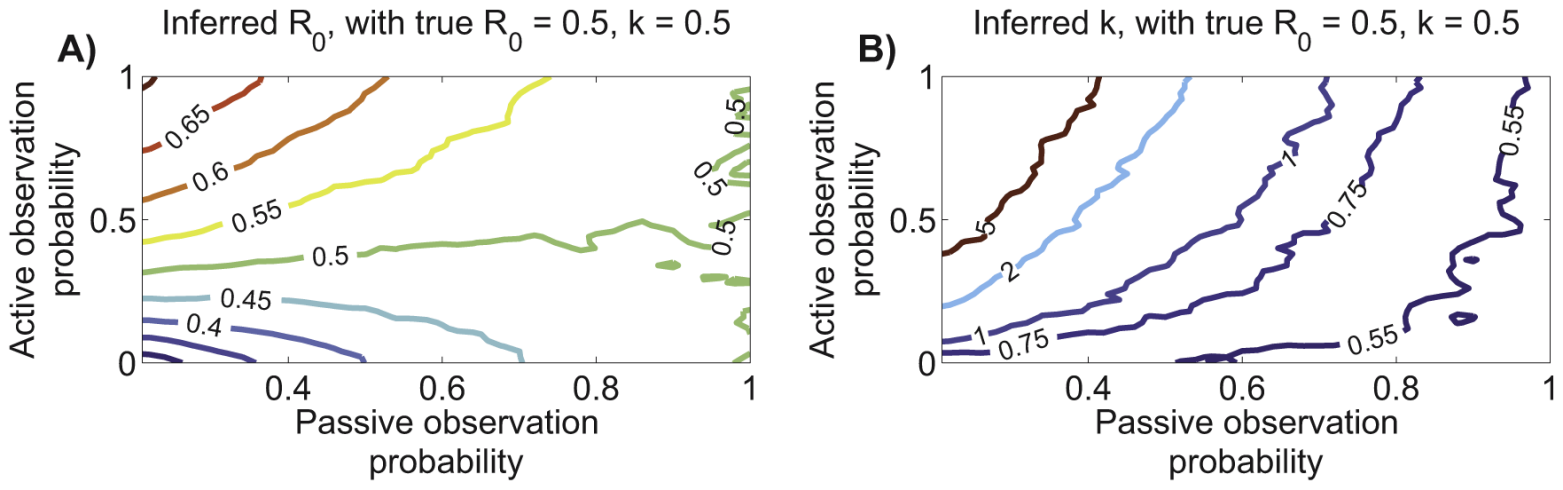
Influence of imperfect observation on 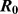 and 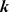 inference. A) The inferred value of 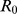 is plotted as a function of the two probabilities we use to model surveillance. Results are based on a simulation of 10,000 chains for a lattice of 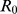 and 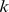 pairs. For visualization purposes, simulation results were smoothed by a one-neighbor moving average. B) Analogous to panel A but for the dispersion parameter.

When the passive observation probability approaches one, essentially all cases are observed and so the inferred 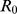 and 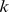 are close to their true value (figure 6). If the passive observation probability is less than one and the active observation probability is low, the average observed size of chains is smaller than the true value, and the 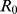 tends to be under-estimated (figure 6A). When the passive observation probability is low but the active observation probability is high, there is a tendency to observe most cases in most of the large chains but to miss many of the small chains entirely. This leads to over-estimation of 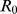.

Imperfect observation tends to cause over-estimation of 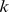, particularly when the passive observation probability is low and the active observation probability is high (figure 6B). This trend arises because the observed fraction of chains that are isolated cases is likely to be under-estimated. Since a high proportion of isolated cases is a hallmark of transmission heterogeneity, inference from data that under-represent isolated cases will be biased toward homogeneity. This implies that when chain size analysis suggests that 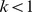 (such as with the 1980s monkeypox data), the conclusion is likely to be a true reflection of heterogeneous transmission dynamics. In contrast, if initial data analysis suggests that transmission is relatively homogeneous, then the possibility that the analysis is impacted by imperfect observation of cases should be considered.

Overall, our observation model suggests that inference of 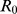 and 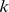 is relatively robust when at least eighty percent of cases are observed. Due to the extensive resources provided for monkeypox surveillance in the 1980s [1], this is likely to have been true for the monkeypox data set we have analyzed. However this level of case detection is unlikely to be attainable for many surveillance programs. An important direction for further work is to correct for imperfect data by incorporating the observation process into the inference framework.

#### Accurate assignment of primary infections is more important than disentangling infection clusters

A key challenge of analyzing chain size data for monkeypox and many other zoonoses is that primary infections are typically clinically indistinguishable from secondary infections. Yet each type of infection represents a distinct transmission process and ignoring this distinction can skew epidemiological assessments. In the context of chain size distributions, this causes a problem because multiple chains can be combined into one cluster. To improve our understanding of how inference of 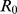 and 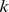 is impacted by how these entangled transmission chains are handled, we compared our initial analysis of monkeypox data to three alternative approaches.

The monkeypox dataset we analyze groups cases in terms of infection clusters rather than transmission chains. Our primary strategy to cope with this limitation was to consider all possible ways that the ambiguous infection clusters could be divided into chains (what we term the combinatorial approach). This effort was greatly facilitated by knowing how many primary cases were present in each infection cluster. We now consider the importance for transmission parameter inference of identifying primary cases correctly. We then consider the additional value of more detailed contact tracing data that allows disentanglement of clusters into individual chains.

**Figure 7:**
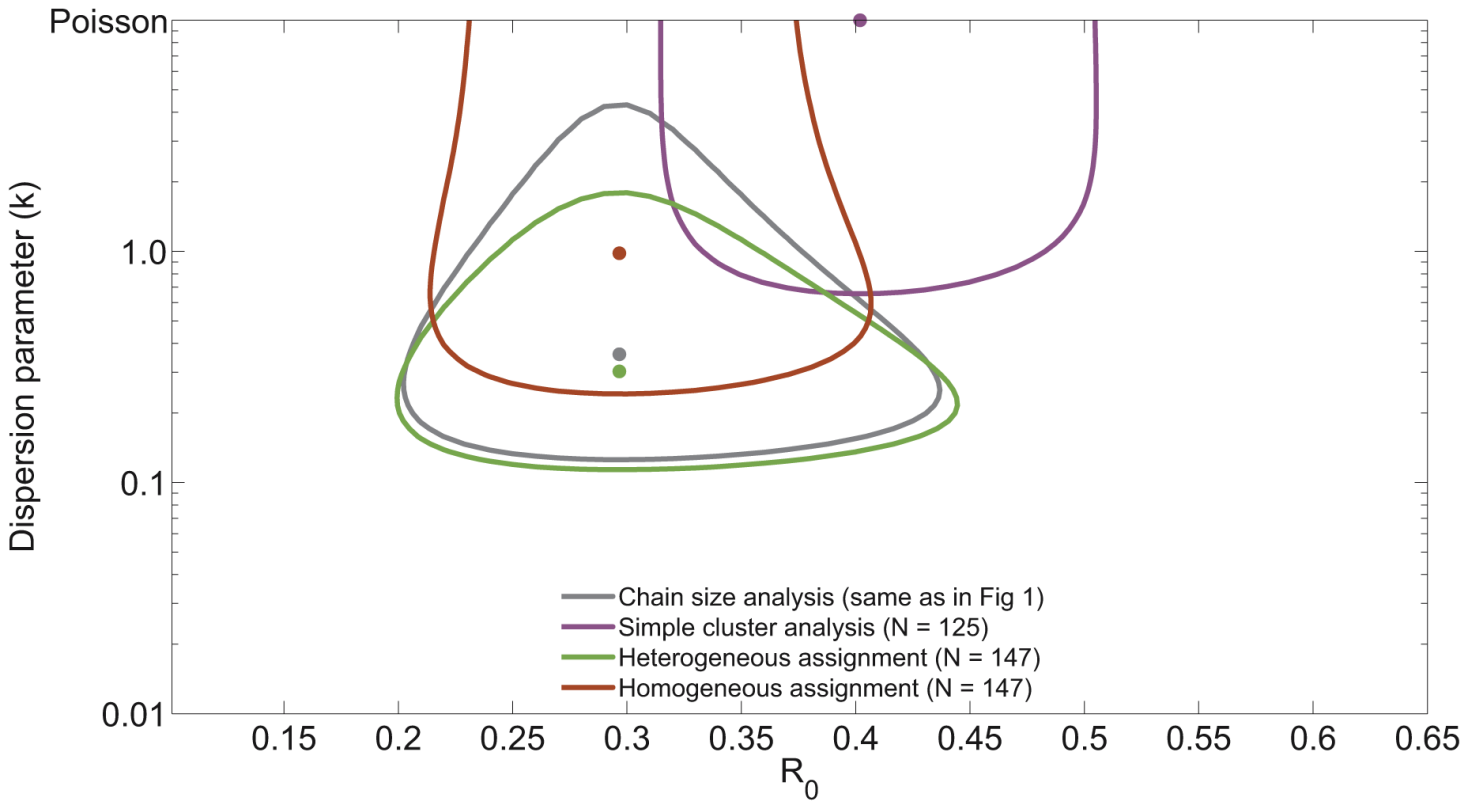
Complications of entangled chains can affect inference. ML estimates of 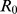 and 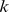 and corresponding 90% confidence regions are when all clusters are treated as chains, and for two approaches to assigning constituent chain sizes for clusters with more than one primary case (details provided in the text). For visual comparison, the contour corresponding to the chain size analysis from figure 1 is replicated.

To assess how clusters identified as having multiple primary infections (equivalent to the presence of ‘co-primary infections’) impact 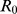 and 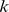 inference, we performed ML inference when the 22 co-primary classifications were ignored and all 125 clusters were treated as single transmission chains (see ‘simple cluster analysis’ in figure 7). The inferred value of 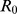 (and its confidence interval) was higher than our original estimate, because ignoring primary infections leads to underestimation of the number of chains, which in turn leads to an increase in the observed average chain size. Further, in contrast to our initial results, the confidence interval for 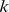 suggests that transmission is unlikely to be more heterogeneous than a geometric distribution. This change arises because treating clusters with co-primary cases as single chains will deflate the apparent frequency of isolated cases, which is a key indicator of transmission heterogeneity.

To determine the importance of disentangling transmission chains fully before performing inference, we considered two methods for dividing infection clusters with multiple primary infections into individual transmission chains (figure 7). Our heterogeneous assignment maximizes the number of isolated cases and thus produces more chains of relatively large size, while the homogeneous assignment minimizes the number of isolated cases and thus produces a higher proportion of intermediate sized chains. The average chain size and corresponding ML estimates of 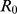 are identical (per equation 12), but the confidence intervals for 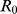 differ slightly depending on the inferred 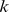 values. Not surprisingly, when clusters are divided as evenly as possible into chains, the ML estimate of 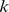 and confidence interval are higher than when clusters are divided in a way that maximizes the number of isolated cases. The ML value based on our initial combinatorial approach (figure 1 and table 1) falls between the ML values obtained using the two assignment procedures. This supports the intuitive conclusion that the true chain assignment is likely a mix of the two extreme assignment algorithms considered.

Only 5 of the 19 clusters containing multiple primary infections had ambiguity with regard to the size of constituent chains. Thus the noticeable difference between the ML estimates of 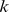 for the homogeneous and heterogeneous chain assignments underscores how the inference of 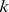 is sensitive to details of infection source assignments. However, the relatively compact confidence region for the combinatorial approach suggests that, in many circumstances, it may not be necessary to disentangle all overlapping transmission chains. In fact, as the homogeneous chain assignment shows, there is a risk that *ad hoc* disentanglement of chains may introduce significant bias in the estimation of 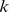. However, for the combinatorial approach to be reliable, it is essential to identify how many cases in each cluster are due to primary infection.

Overall, our analysis of monkeypox data highlights how inference of transmission parameters from chain size data can be complicated when infection clusters may contain multiple primary infections. More generally, the challenge of properly differentiating primary from secondary infections is of fundamental importance for analysis of stuttering zoonoses. Even when well-trained surveillance teams are on site to assess transmission pathways, it may be impossible for them to decide between two equally likely infection sources. For instance, it can be difficult to decide if a mother contracted monkeypox because she cared for an infected child or because she contacted infected meat (in the same contact event as the child, or a later one). The theory presented here forms a foundation for further research on infection source assignment and its relationship to underlying transmission mechanisms. Future investigations can leverage existing methods of source assignment developed for supercritical diseases, which utilize various epidemiological data such as symptom onset time, risk factor identification and pathogen genetic sequence data [32]–[34]. These types of theoretical developments, combined with strong collaborative ties between field epidemiologists and modelers, would likely expand the use of existing epidemiological data and improve resource allocation for future surveillance efforts.

### Model limitations

Several of our modeling assumptions deserve further exploration. In particular, the assumption that transmission can be described by independent and identical draws from a negative binomial offspring distribution is a simplification of some forms of transmission heterogeneity. For example, if heterogeneity is driven largely by population structure, such that susceptibility and infectiousness are correlated, then the relation between 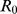 and heterogeneity can differ from what is represented in our model [35]. Specific scenarios that can give rise to such correlations include the existence of clustered pockets of susceptible individuals, impacts of coinfection or immunosuppressive conditions, or transmission heterogeneity that arises chiefly from variation in contact rates rather than variation in the amount of virus shed [36]–[38]. This issue is especially relevant for preventable diseases such as measles, because large outbreaks in developed countries are often associated with particular communities in which vaccine refusal is common [16], [39]. Local depletion of susceptible individuals, which can even occur within a household, can also impact the estimation of 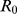 and 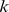. By diminishing the possibility of large outbreaks, the depletion of a susceptible population is likely to decrease estimates of 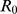 and increase estimates of 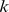. We hope that our use of a likelihood function that combines 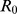 and transmission heterogeneity will facilitate future work that addresses these modeling challenges in a self-consistent manner.

### Conclusion

Data acquisition is often the limiting factor for assessing the transmission of subcritical diseases that pose a threat of emergence. Our findings can assist future surveillance planning by drawing attention to the utility of chain size data when contact tracing data are too difficult to obtain. We have shown that both 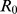 and the degree of transmission heterogeneity can be inferred from chain size data, and have demonstrated that chain size data can give equivalent power to contact tracing data when deciding if 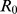 has changed over time. In fact, even knowledge of the largest chain size alone can be helpful for monitoring change in 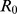, provided that the degree of transmission heterogeneity has been reliably measured. Conversely, we have demonstrated that inaccurate assumptions about transmission heterogeneity can lead to errors in 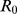 estimates and possible false alarms about increased transmission. We have also found that inference can be accomplished when transmission chains are entangled into infection clusters, provided that the number of primary infections in each cluster is known. For the particular case of human monkeypox, our findings support previous analyses that have identified substantial transmission heterogeneity, but conclude that endemic spread would only be possible if there is significant demographic change or viral adaptation to enable greater human-to-human transmissibility. Since a mechanistic understanding of transmission dynamics is important for quantifying the risk of emerging diseases and predicting the impact of control interventions, we hope our findings will assist in providing robust epidemiological assessments for relevant public health decision-making.

## Methods

### Monkeypox data

We analyzed previously reported data describing monkeypox cases identified between 1980–1984 in the Democratic Republic of Congo (formerly Zaire) [1]. These data were collected in order to assess the potential of monkeypox to emerge as an endemic human pathogen in the wake of smallpox eradication. Contact tracing and subsequent analysis by epidemiological teams classified each identified cases as a primary case, arising from animal-to-human transmission, or a secondary case, arising from human-to-human transmission. The data set consists of 125 infection clusters [26], [27]. Most clusters contained just one primary case and thus constituted a single transmission chain. However nineteen of the clusters had overlapping transmission chains, because contact tracing revealed they contained more than one primary case.

The raw cluster data for monkeypox was obtained from table 1 of [26]. Our baseline inference of transmission parameters is based on considering all the possible ways this cluster data can be separated into individual transmission chains. To explore the specific impact of entangled transmission chains on the inference of transmission parameters, we also investigated the impact of three approaches of using the cluster size data to assign an explicit chain size distribution (table 4). In the ‘simple cluster analysis’ approach, we treat all clusters as though they were a complete stuttering chain and ignore the complications of multiple primary infections. The other two approaches use different algorithms to divide the clusters that have multiple primary infections into constituent chains. In our ‘homogeneous assignment’ distribution, clusters were divided as evenly as possible. For example, a cluster of total size four with two co-primary cases is tabulated as two chains of size two. Meanwhile, our ‘heterogeneous assignment’ distribution maximized the number of isolated case counts when disentangling clusters. For this distribution, a cluster of size four with two co-primaries is tabulated as a chain of size one and a chain of size three.

**Table 4:** The number of transmission chains tabulated by size (i.e. total number of cases) for three different assignment algorithms.

Chain size	Simple cluster analysis	homogeneous assignment	heterogeneous assignment
1	84	114	120
2	19	16	7
3	11	11	12
4	5	2	3
5	2	2	3
6	4	2	2

### Offspring distribution

We analyze the transmission dynamics of stuttering chains using the theory of branching process [22], [40], [41]. The key component of this theory is the probability generating function, 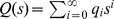 of the offspring distribution. This function describes the probability distribution for the number of new infections that will be caused by each infected case. The probability that an infected individual directly causes 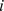 infections is 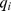, and hence the probability that an individual is a dead-end for transmission is 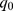. Subject to the standard assumption that transmission events are independent and identically distributed, 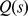 contains all the information needed to determine the size distribution of stuttering chains.

The choice of offspring distribution is important because it defines the relationship between the intensity and heterogeneity of transmission. We adopt a flexible framework by assuming secondary transmission can be characterized by a negative binomial distribution with mean 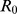 and dispersion parameter 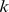. The corresponding generating function, valid for all positive real values of 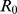 and 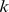, is [24]

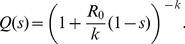
(1)


A key advantage of using a two-parameter distribution over a one-parameter distribution (such as the geometric or Poisson distribution) is that modulating 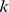 permits the variance to mean ratio, 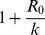, to range from one to 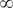 without any change in 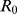. Further, the geometric and Poisson distributions are conveniently nested cases of the negative binomial distribution when 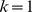 and 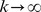 respectively.

### Simulations

All simulated chains start with a single primary infection. Then the number of first generation cases is decided by choosing a random number of secondary cases according to a negative binomial distribution with mean 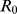 and dispersion parameter 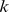. For each case in the first generation (if any exist), a new random number is chosen to determine how many consequent second generation cases there are. This is repeated until the stuttering chain goes extinct. Since our focus is on 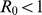, all simulated chains eventually go extinct. Simulated contact tracing data consisted of the individual transmission events that produce simulated chain size data.

To simulate imperfect observation, we first simulated a set of true transmission chains, then simulated whether each case would be observed according to the passive observation probability. Finally, for chains where at least one case was detected passively, we simulated which additional cases were observed according to the active observation probability.

All calculations and simulations are performed with Matlab 7.9.0. Code is available in Text S2.

### Stuttering chain statistics

The next two subsections derive the average size and variance of the distribution. As a by-product, we obtain a first order moment estimator for 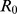 and a second order moment estimator for 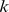. We will see that the first order moment estimator of 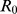 exactly matches the ML value of 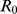. This finding provides a simple relationship between observed data and 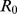 inference.

#### Average size of stuttering chains

Since the average number of cases per generation declines in a geometric series when 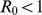, the average stuttering chain size, 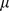, is simply [21], [41], [42]

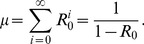
(2)


This relationship can be inverted to obtain the first moment estimator for 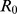 based on the observed mean chain size, 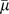,
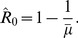
(3)


An alternative expression for 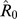 can be obtained for a data set encompassing numerous chains by letting 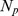 and 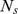 denote the number of primary and secondary cases, respectively. Then since 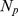 is the total number of chains and 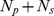 is the total number of cases, 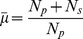. Therefore,
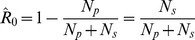
(4)which is the fraction of all observed cases due to secondary transmission, as noted previously [21].

#### Coefficient of variance for the offspring and chain size distributions

The coefficient of variation (COV) provides quantitative perspective on the relationship between 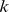 and observation of cases. The variance of the negative binomial distribution is given by 
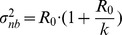
. Therefore the COV for the offspring distribution, 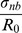, is
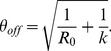
(5)


Meanwhile, branching process theory shows that the variance of the chain size distribution is 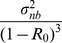 when 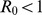 [41], [42]. Therefore the COV for the chain size distribution is,
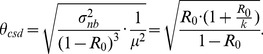
(6)


**Figure 8:**
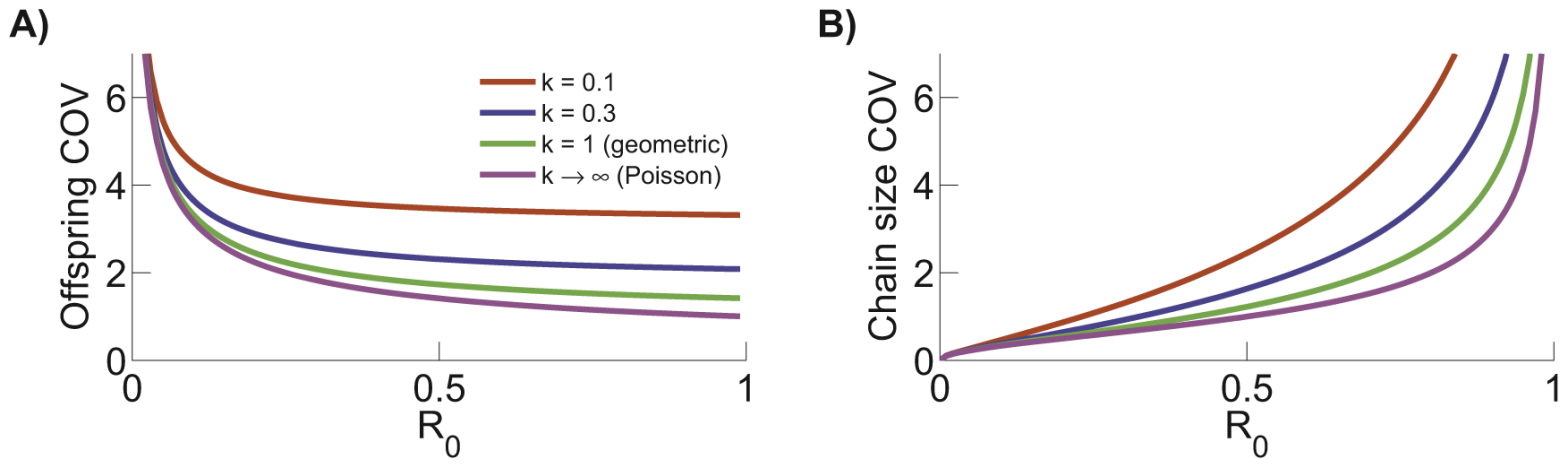
Coefficient of variation for offspring and chain size distribution. The COV for the offspring distribution (i.e. the distribution for the number of transmission events caused by each case, panel A) and chain size distribution (panel B) are both a function of 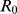 and 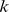.

The COV of the negative binomial offspring distribution increases as 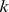 decreases (figure 8A), reflecting the rise in transmission heterogeneity [24], [43]. The COV of the chain size distribution also increases as 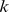 decreases (equation 6, figure 8B). In contrast to the COV of the offspring distribution, for a given value of 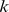, the COV of the chain size distribution increases as 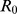 increases. This is due to stochastic variation, which gets amplified for longer chains as 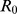 rises.

Equation 6 can be inverted to obtain a second moment estimator for 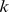 based on the observed coefficient of variation, 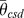, and the inferred 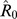,
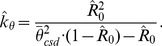
(7)The 2nd moment estimator of 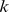 does not always provide valid inference of 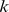 because the denominator can be negative. Because this circumstance arises when the chain size variance is particularly small, we interpret it as corresponding to a Poisson offspring distribution since this is the most homogeneous distribution allowed by the negative binomial model.

### Size distribution of stuttering chains

Beyond determining the relationship between 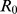, 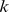, 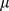 and 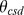, our assumptions about the transmission process allow us to use branching process theory to characterize the complete size distribution of stuttering chains [23], [40]–[42]. Let 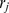 be the probability of a transmission chain having overall size 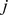. If one defines 
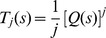
, then [44],
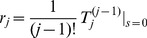
(8)where 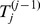 is the 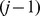
th derivative of 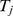. See the supporting text (Text S1) for a derivation of this formula that develops intuition for the specific application to disease transmission. In particular, the supporting text explains the validity of equation 8 for both 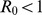 and 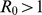, which extends recent findings of Nishiura et al. [23].

Based on equation 1 the formulae for 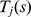 and 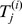 are,
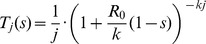


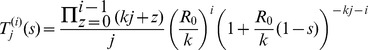
where the latter formula was derived by induction. Substitution into equation 8 gives,
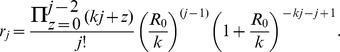



Noting that the Gamma function 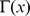 satisfies 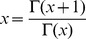 and that 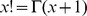 for integer 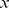, we can rewrite the last formula as
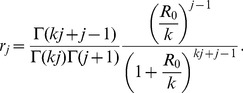
(9)This equation matches the relation derived by Nishiura et al. for the specific case of 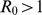 [23]. This relationship was verified by using a stochastic simulation model to simulate many stuttering chains as described above (data not shown).

Equation 9 forms the basis of interpreting chain size distribution data because it provides the probability that a randomly chosen stuttering chain has a size 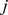. However, from the perspective of considering how chain size observations reflect overall disease burden, it is also helpful to consider the probability, 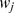, that a randomly chosen case is in a stuttering chain of size 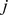. This ‘weighted’ probability density is obtained by scaling each 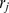 by 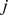 and then renormalizing. Accordingly,
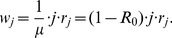
(10)


**Figure 9:**
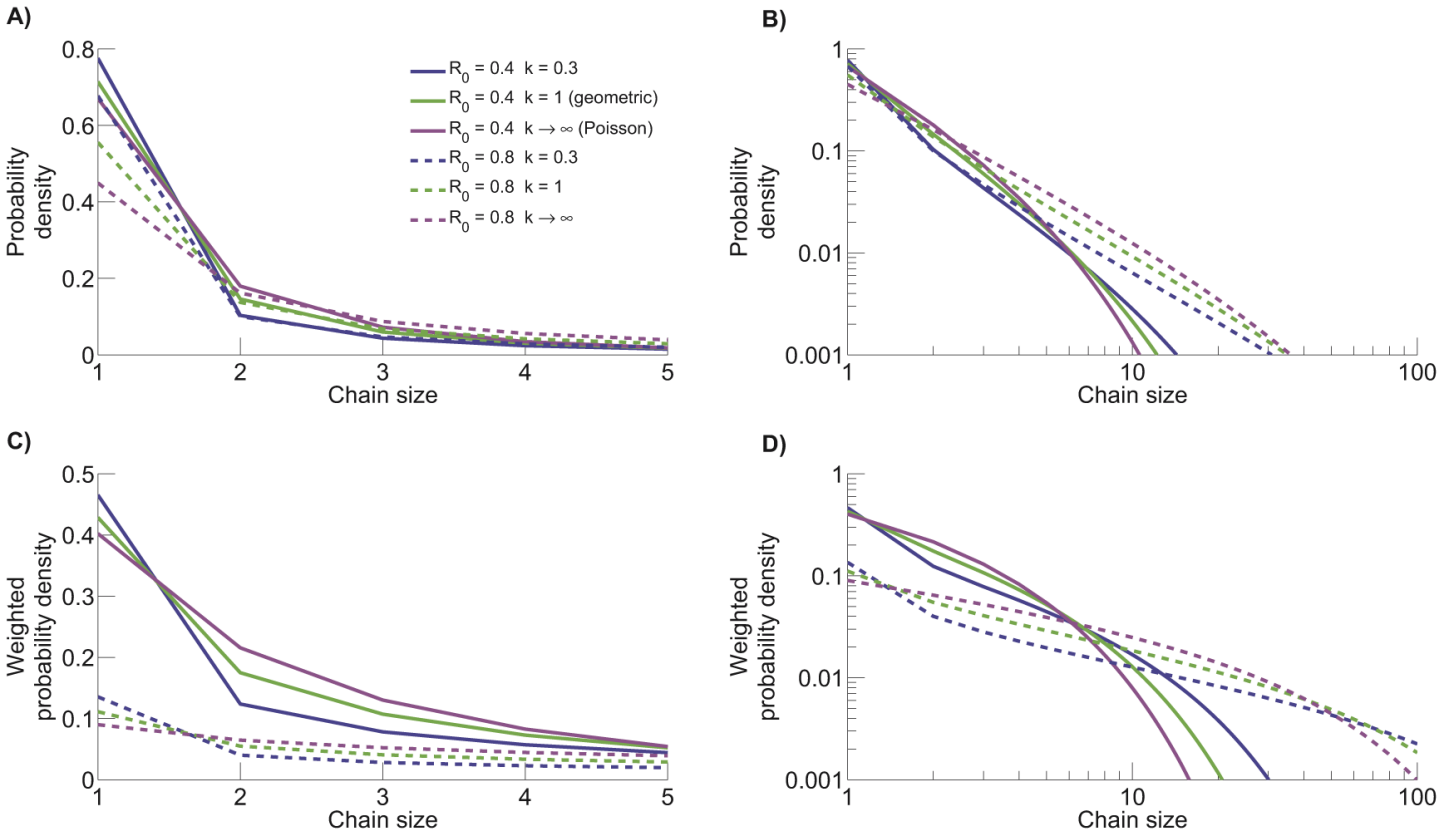
The size distribution of stuttering chains varies as a function of 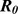 and 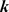. A) The probability distribution for chain sizes for various parameter choices, when transmission is described by a negative binomial offspring distribution. B) Same as panel A but with logarithmically scaled axes, to highlight lower frequencies and larger chain sizes. C) The weighted probability density for the same 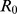 and 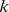 pairs given in panel A. D) Same as C with logarithmically scaled axes. The legend in panel A applies to all panels.

For a given value of 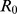, decreasing 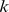 leads to both a higher number of isolated cases and a higher number of large stuttering chains (figure 9). Meanwhile, the homogeneous Poisson offspring distribution maintains the highest probabilities for intermediate sized stuttering chains (seen most clearly in figure 9D), Thus, branching process theory provides an analytical foundation for prior computational results showing that greater transmission heterogeneity results in a higher frequency of relatively large stuttering chains [24], [25], [29], [43], [45]. Of particular interest, the fraction of stuttering chains that consist of a single isolated case is substantial for all parameter sets considered. Meanwhile, the weighted probability density shows that the probability of a case occurring as an isolated case can be significantly less than the probability of a randomly chosen stuttering chain having size one.

### Maximum likelihood estimation of 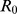 and 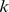


We employ maximum likelihood estimation for 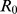 and 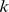 inference because it is asymptotically unbiased and maximally efficient (i.e. there is minimum sampling variance). To implement ML estimation for 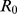 and 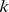 using stuttering chain size distribution data, we let 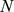 denote the total number of stuttering chains in a given dataset, and 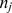 represent the number of chains with size 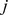. Then the likelihood, 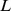, of the data set is,
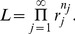
(11)


The ML estimate of 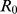 and 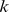 is found by maximizing the log-likelihood function with respect to both parameters. The maximum occurs when 
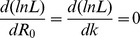
. Focusing on finding the ML estimate for 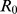, one finds,
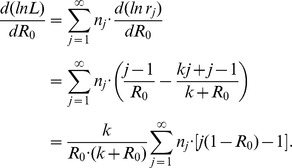
Then since the total number of chains is 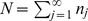 and the observed average chain size is 
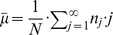
,
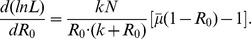



Solving for 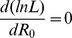 gives,
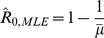
(12)which is identical to the first moment estimator 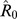 given by equation 2.

The ML calculation for the dispersion parameter, 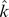, is not analytically tractable and 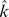 depends on 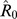. Thus, 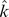 is obtained by computational optimization of the log likelihood. Since the limits 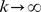 and 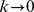 lead to convergence difficulties, we set lower and upper limits of 0.00001 and 1000 for 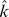. This lower bound for 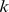 is well below the range needed to infer biologically relevant values of 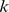 and the upper bound for 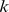 is essentially equivalent to a Poisson distribution. We cannot attempt 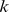 inference when a simulated data set has no secondary transmission (implying 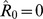). Therefore these data sets, which occasionally occur when both 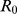 and 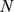 are low, were discarded from our simulation-based characterization of 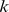 inference.

#### Combinatorial method for maximum likelihood estimation of 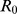 and 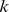 for monkeypox clusters

As mentioned, some of the monkeypox infection clusters could not be unambiguously divided into constituent chains. For our baseline ML inference of 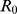 and 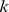 (figure 1 and table 1), we approach this ambiguity by considering all possibilities of chains that could give rise to clusters of the observed size. For instance, the probability that an infection cluster having two primary infections has an overall size of four is,
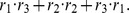
To conduct inference of 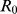 and 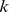 these combinatorial terms were included in the product of equation 11.

#### Contact tracing method for maximum likelihood estimation of 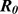 and 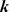 for monkeypox

Contact tracing investigations yield direct information about how many infections are caused by each infectious case. By analogy to equation 11, the likelihood of contact tracing data can be written as,
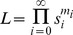
(13)where 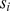 is the probability that a case will directly cause 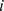 infections and 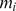 is the number of cases that directly cause 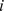 infections. For our model, 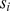 is the probability density of a negative binomial distribution,
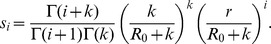
Although full contact tracing data are unavailable for monkeypox in the 1980s, much of it can be reconstructed from the tabulation of monkeypox cases in which the number of cases is noted for each generation of each cluster (table 1 of [26]). As in the case of infection clusters with multiple primary infections, there is some ambiguity in the contact tracing data for 11 of the 209 cases when it is only known that a set of cases lead to one or more infections. However, it is straightforward to consider the probability for each of the possible combinations and incorporate their sum as a factor in equation 13. This combinatorial approach was used to create figure 1 and table 1.

### Measuring the performance of 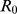 and 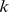 inference

To study the precision and accuracy of our ML approach, we simulated many data sets for a range of values of 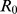, 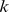 and 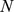. We inferred the ML values of 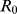 and 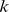 from the simulated data, and compared these values to the true values used in the simulation.

#### Error of 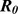 and 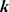 inference

We use two metrics to summarize the error in inferred values of 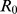. The first metric is the root mean square relative error, defined as
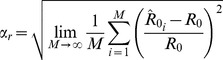
(14)where 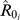 is the ML value of 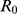 for a simulated dataset 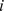 which had true parameter values 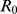, 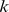 and 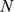. In practice, the limit is taken to a reasonable number of simulations, 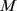, based on convergence of 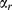 (we typically set 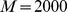).

Another useful metric for characterizing 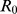 inference is the root mean square absolute error defined as
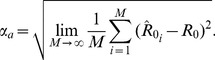
(15)


Since the relative error scales with 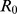 it can be particularly useful in assessing the significance of small differences between 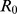 values when secondary transmission is quite weak. Meanwhile, as explained below, the absolute error is useful for decomposing the source of 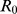 measurement uncertainty into bias and unavoidable stochastic randomness.

Since the coefficient of variation of the negative binomial distribution is a function of 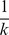, the effect of changing 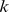 by a fixed amount is much greater when 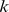 is small than when 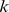 is large. Therefore we choose to measure the error as the difference in the reciprocals of the inferred and true 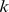, because this leads to more consistent interpretation of inference results. The convention of using the reciprocal transform for inference on 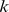 is well established in the biostatistics literature on negative binomial inference [29], [30]. We define the root mean square error of 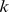 as,
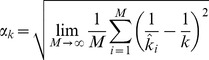
(16)where 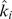 is the ML estimate of 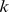 for the 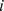
th dataset. Whenever 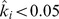, it is replaced by 0.05 in this calculation to avoid numerical instabilities arising from small denominators. The threshold of 0.05 was chosen because it is close to, but below the observed range for 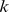 in infectious disease transmission data [24].

#### Bias of 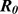 inference

The inference error for 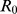 contains contributions from estimator bias and from the inherently random nature of the processes generating the data. The bias of 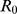 inference is given by
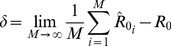
(17)for fixed 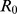, 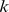 and 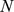. The contribution of randomness is summarized by the standard deviation of the ML values of 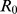 associated with a set of simulation parameters, 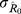. The two sources of error add in quadrature to form the root mean square absolute error,
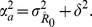
If the bias were eliminated from the 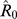 estimator, then the error would simply be 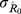. Therefore, the fractional reduction of the absolute error in 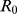 inference that would be possible with optimal bias correction is
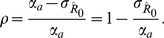
(18)


#### Confidence intervals

We use likelihood profiling to determine the confidence intervals for inferred values of 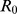. More specifically, for a given dataset let 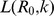 denote the likelihood for particular values of the parameters 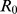 and 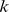. Then define 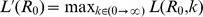. In addition, let 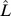 denote the likelihood for the ML estimates of 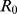 and 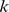. Then the endpoints of the confidence interval corresponding to a confidence level 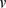 are obtained by finding the two values of 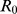 that solve,
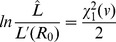
(19)where 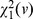 denotes the inverse of the chi-square cumulative distribution function for one degree of freedom [31].

Our approach does not put any explicit constraints on the value of 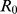, but equation 12 will always produce a ML estimate satisfying 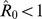, implying that subcritical transmission is likely when all observed chains are self-limited. However, if 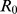 exceeds one and the number of observations is small, all observed chains may be self-limited due to stochastic extinction. Therefore, 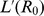 is continuous across the critical value of 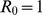 and the upper limit of the 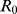 confidence interval can exceed one.

To determine the associated confidence interval for 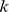 inference, we define 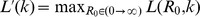. Then the confidence interval endpoints are the two values of 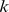 that solve
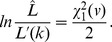
(20)


The two-dimensional confidence regions corresponding to a confidence level of 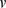 are determined by finding the 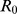 and 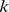 pairs that satisfy,
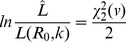
(21)where 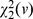 is the inverse of the chi-square cumulative distribution function for two degrees of freedom.

To test the accuracy of the ML confidence intervals, we use simulated data to determine the coverage probabilities of the univariate confidence intervals for 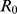 and 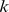. The coverage probability equals the proportion of simulated data sets for which the ML confidence interval includes the true value of the relevant transmission parameter. For example, the 90% coverage probability for 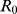 inference is determined by counting the fraction of simulations for which
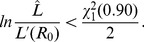
(22)


### Inference of 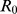 from different types of epidemiological data

#### When combined with chain size data, additional data on the generation of extinction do not change the ML value of 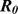


Prior research has shown that the distribution of the number of transmission generations before extinction for a set of stuttering chains can be used to infer 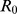 [22]. Consistent with this prior analysis, we find that 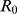 inference can be achieved using just the generation-of-extinction distribution in a ML framework, but that the chain size distribution produces a more precise 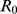 estimate for a given number of chains in the data set (simulation-based results not shown). Here we extend this result by showing that joint knowledge of chain size and the number of generations before extinction does not change the ML estimate of 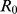 from the value obtained from the chain size distribution alone.

The joint likelihood of a chain having size 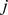 and lasting 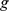 generations is
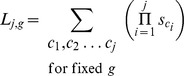
where 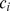 represents the number of offspring that individual 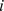 has, 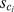 is the probability an individual has 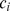 offspring, and the sum is over all possible offspring combinations that form a transmission chain of size 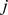 having 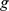 generations. For a negative binomial offspring distribution,
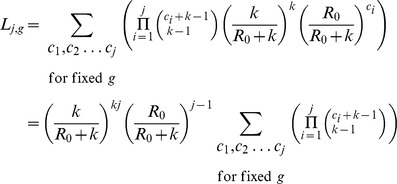
where we have utilized 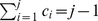 because every chain of size 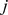 has one primary and 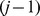 secondary infections. If we define 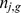 to be the observed number of chains of size 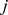 and having 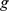 generations, then the overall likelihood of a dataset is,
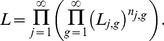
Setting 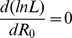,
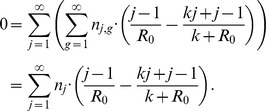



Since this now overlaps with the derivation of equation 12, we find that the new ML value for 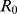 is identical to our initial estimate, 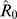. Thus when there is perfect case detection, knowledge of the number of generations in a chain does not change the ML value for 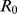. When case detection is imperfect it may be that combined use of chain size and generation of extinction data could yield more precise estimates than chain size data alone.

#### Compared to chain size data, contact tracing does not change the ML value of 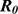


We now assume that we have complete contact tracing data, meaning that for every infected individual we know exactly how many individuals they subsequently infected. The likelihood is given by equation 13 and solving 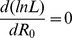 to determine the ML value yields,
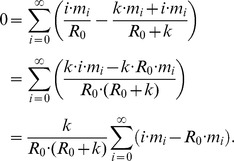



This means the ML estimate of R_0_ based on contact tracing data is:
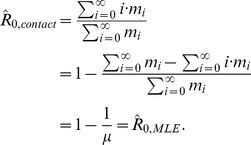



Thus, when estimating 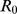 for subcritical (i.e. 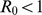) transmission with perfect case detection, contact tracing data does not change the ML value of 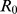 from that determined from the chain size distribution.

### Monitoring changes in 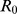


To determine whether two data sets on chain size distribution correspond to statistically distinct values of 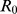, we performed a likelihood ratio test. First, we combined all data together and calculated the likelihood, 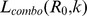, for a single pair of 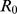 and 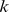 values. Then we computed a second likelihood, 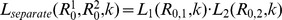 where 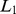 and 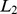 are the likelihoods for each set of data and each of these likelihood functions has its own 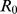 parameter. We kept 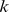 constant for both sets of data in order to focus on whether there is a statistically significant change in 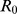. Because 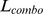 is nested within 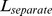 (equality occurring when 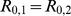), we apply the likelihood ratio test with a 95% confidence interval cutoff to determine whether a second 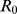 parameter is justified [31].

For figure 2A, 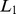 was equal to the combinatorial likelihood calculation for the 1980s contact tracing monkeypox data. Meanwhile, 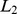 was calculated from simulation data in which 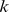 was fixed at the ML value for the 1980s data. We conducted one thousand simulations for each value of 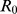, and computed the proportion of simulations for which a second 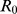 parameter was supported by the likelihood ratio test. For figure 2B, a similar set of calculations was performed, except that the ML values were obtained by fixing 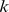 at either 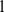 or 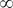 in the calculation of 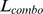 and 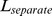.

The results presented in table 2 concerning the probability that a change in 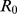 is erroneously detected were determined by simulating two sets of chain size data using the ML values for 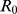 and 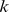 from contact tracing data for human monkeypox the 1980s (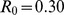, 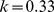). Likelihood scores were calculated for the stated values of 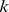, and the likelihood ratio test was used to assess whether 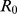 had changed significantly between the two data sets. Because a 95% confidence level was used for this test, a statistical difference is expected just 5% of the time. Higher frequencies of falsely detecting a change in 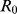 correspond to artifacts of the inaccurately narrow confidence intervals obtained when transmission heterogeneity is under-estimated.

### Determining chain size cutoffs

The probability that a chain has a size less than 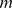 is the sum of the individual chain size probabilities, 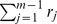. The probability, 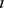, that 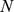 chains all have a size less than 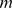 is the product of the individual probabilities for each chain to have a size less than 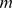:
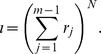

Figure 3 plots the first value of 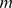 for which 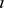 exceeds the indicated probability threshold.

## Supporting Information

Figure S1
**Conceptualizing the combinatorics of stuttering transmission chains.** A) Example of a stuttering transmission chain. The unique offspring sequence for this stuttering chain is 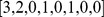. B) Representation of an invalid transmission sequence. The black line shows the cumulative reproduction number, 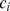, as defined in the text for transmission sequence 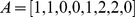. The blue line corresponds to 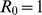 for all cases and marks an extinction boundary. Thus 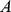 is an invalid transmission chain because it crosses the blue line after the third case. The green line graphically represents the minimization of the number of extant infectors, 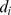, and shows that the corresponding valid transmission sequence should start with the fifth individual. C) Representation of corresponding valid transmission sequence. Analogous to panel B except that the fifth cyclic permutation of 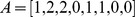 is plotted. Now the green and blue lines overlap showing that the proper start point is with case one and the stuttering chain goes extinct only after all individual infections have been accounted for.(TIF)

Text S1
**The supporting text derives the relationship between the offspring distribution and the size distribution of transmission chains.** The derivation holds for both 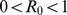 and for 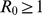 **.**
(PDF)

Text S2 **Matlab code for two key functions is provided. One function shows how we simulate the transmission and observation process models presented in this manuscript. The other function shows how the probability density can be analytically calculated for a given set of transmission parameters.** This probability density can then be used to calculate the likelihood function used in all of our ML analyses.
(PDF)
