# Supplementary figures and images for: Inference of R 0 and Transmission Heterogeneity from the Size Distribution of Stuttering Chains
Source: PLoS Comput Biol. 2013 May 2;9(5):e1002993. doi: 10.1371/journal.pcbi.1002993 (PMC3642075; doi:10.1371/journal.pcbi.1002993)

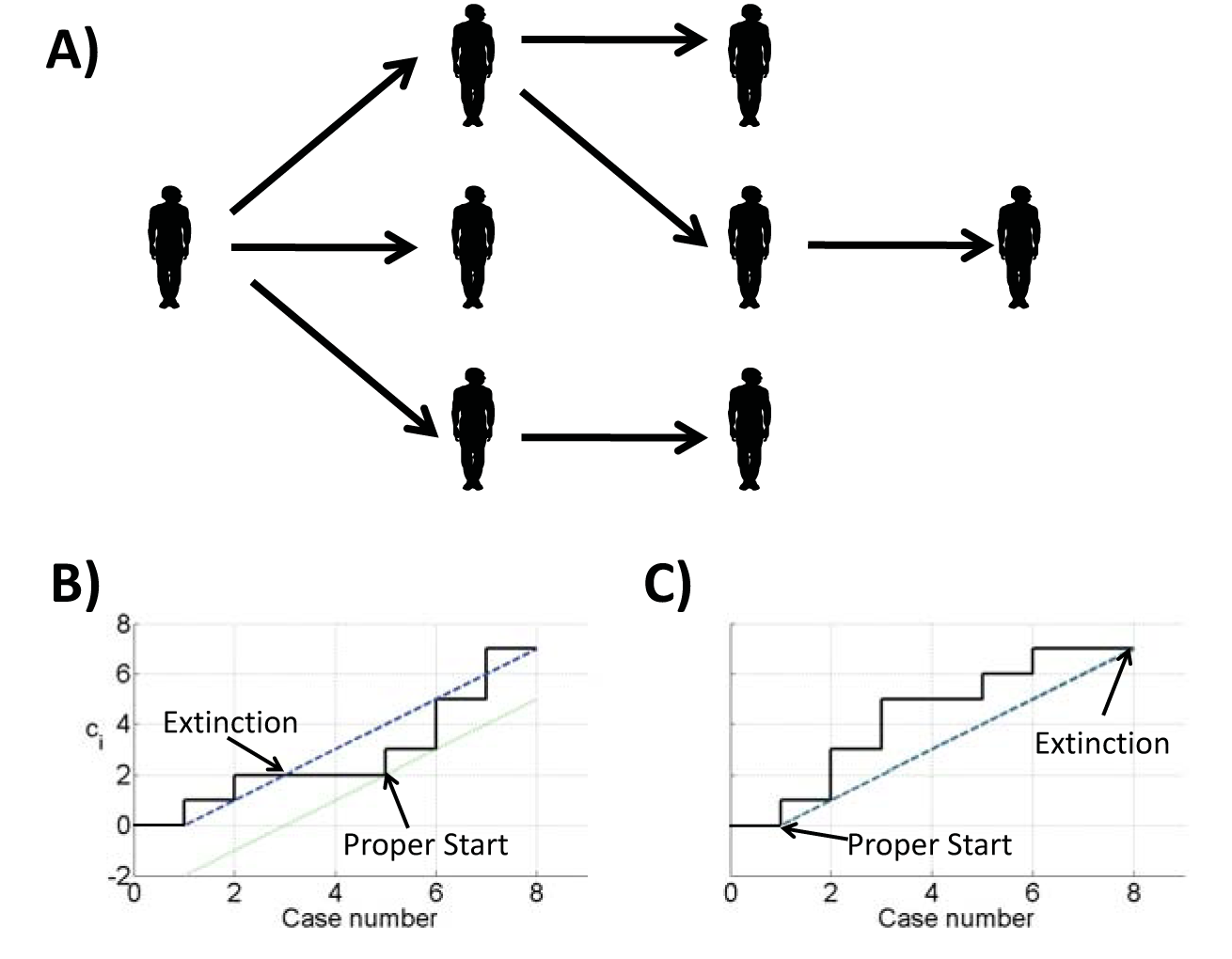

Supplement: Figure S1 — Conceptualizing the combinatorics of stuttering transmission chains. A) Example of a stuttering transmission chain. The unique offspring sequence for this stuttering chain is . B) Representation of an invalid transmission sequence. The black line shows the cumulative reproduction number, , as defined in the text for transmission sequence . The blue line corresponds to for all cases and marks an extinction boundary. Thus is an invalid transmission chain because it crosses the blue line after the third case. The green line graphically represents the minimization of the number of extant infectors, , and shows that the corresponding valid transmission sequence should start with the fifth individual. C) Representation of corresponding valid transmission sequence. Analogous to panel B except that the fifth cyclic permutation of is plotted. Now the green and blue lines overlap showing that the proper start point is with case one and the stuttering chain goes extinct only after all individual infections have been accounted for. (TIF) [file pcbi.1002993.s001.tif]
