# Supplementary material for: Inference of R 0 and Transmission Heterogeneity from the Size Distribution of Stuttering Chains
Source: PLoS Comput Biol. 2013 May 2;9(5):e1002993. doi: 10.1371/journal.pcbi.1002993 (PMC3642075; doi:10.1371/journal.pcbi.1002993)
Supplement: Text S1 — The supporting text derives the relationship between the offspring distribution and the size distribution of transmission chains. The derivation holds for both and for . (PDF) [file pcbi.1002993.s002.pdf]

Text S1: Relating the offspring distribution to the size distribution of transmission chains

Recall that the coefficient q_i of the offspring generating function $Q(s) = \sum_{i=0}^{\infty} q_i s^i$ specifies the probability that an infected individual will cause i subsequent infections. Therefore, the kth coefficient of $[Q(s)]^2$, equal to $\sum_{i=0}^{k} q_i q_{k-i}$, is the probability that two individuals cause a total of k infections. By induction, it follows that the kth coefficient $[Q(s)]^n$ is the probability that n individuals cause a total of k infections. Meanwhile, for every transmission chain of size j, there are j individuals that cause $j-1$ infections. However, the probability of j individuals causing $j-1$ infections is not the same as the probability, r_j, that a stuttering chain has size j, because in the latter case the order of the infections matter. For example, if the index case has no offspring then there will not be a chain of size j, even if the remaining $j-1$ individuals cause $j-1$ infections.

Fortunately, there is a redundancy that allows us to relate r_j to the probability that j individuals have $j-1$ offspring. First note that there is a one-to-one mapping between a sequence of j non-negative integers that sum up to $j-1$ and the way in which j individuals can have $j-1$ offspring. Let us represent one such offspring sequence, A, as $a_1 a_2 \ldots a_j$ meaning that individual i has a_i offspring. Meanwhile, every stuttering chain of size j can be described by an offspring sequence $B = b_1 b_2 \ldots b_j$ of non-negative integers (figure S1A). This is accomplished by setting b_1 equal to the number of offspring of the primary case (i.e. first generation), the next b_1 numbers equal to the number of offspring for individuals in the second generation, the next numbers equal to the number of offspring for individuals in the third generation and so forth, until the stuttering chain ends.

Theorem 1: For every offspring sequence $A = a_1 a_2 \ldots a_j$ that satisfies $\sum_{i=1}^{j} a_i = j-1$, exactly one of the cyclic permutations

$$\begin{aligned}
A'_1 &= a_1 a_2 \ldots a_{j-1} a_j, \\
A'_2 &= a_2 a_3 \ldots a_j a_1, \\
&\ldots \\
A'_{j-1} &= a_{j-1} a_j \ldots a_{j-3} a_{j-2}, \\
A'_j &= a_j a_1 \ldots a_{j-2} a_{j-1}
\end{aligned}$$

corresponds to a valid transmission sequence.

Proof: For an offspring sequence $B = b_1 b_2 \ldots b_j$ let the cumulative reproduction number, $c_i^B = \sum_{l=1}^{i} b_l$, denote the number of infections caused by individuals $1, 2, \ldots i$. Then for B to be a valid transmission sequence, the necessary and sufficient conditions are,

1. $c_j^B = j-1$
2. $c_i^B \geq i$ for $i < j$.

The first condition assures a stuttering chain of size j has exactly one primary infection and $j-1$ secondary infections. The second condition assures that the stuttering chain does not go extinct before reaching a size of j.

Note that for our definition of the offspring sequences, A'_i, condition 1 is always satisfied. Thus to complete our proof we first show there exists a k such that A'_k satisfies condition 2 and then we show k is unique. Define $c_0^A = 0$ and let k' equal the value of $i \in \{1, 2, \ldots j\}$ that results in the first occurrence of the global minimum of the number of extant infectors, $d_i \equiv c_{i-1}^A - i$. As illustrated conceptually in figures S1B and S1C, we will show $A'_{k'}$ is the unique cyclic permutation of A that is a valid transmission sequence. To prove existence, consider four possible value ranges for k' and i.

Case 1: $k' = 1$ and $i < j$. Then $d_{i+1} \geq -1$ because $d_1 = -1$ and so

$$\begin{aligned} c_i^A &= (c_i^A - i - 1) + i + 1 = d_{i+1} + i + 1 \\ &\geq i \end{aligned}$$

Case 2: $k' > 1$ and $i \leq j - k'$. Here, $d_{k'+i} \geq d_{k'}$ and so

$$\begin{aligned} c_i^{A'_{k'}} &= c_{k'+i-1}^A - c_{k'-1}^A \\ &= (c_{k'+i-1}^A - k' - i) + k' + i - (c_{k'-1}^A - k') - k' \\ &= d_{k'+i} - d_{k'} + i \\ &\geq i \end{aligned}$$

Case 3: $k' > 1$ and $i = j - k' + 1$. Noting that $d_{k'} \leq d_1 - 1 = -2$

$$\begin{aligned} c_i^{A'_{k'}} &= c_{k'+i-1}^A - c_{k'-1}^A \\ &= c_j^A - c_{k'-1}^A \\ &= j - 1 - (c_{k'-1}^A - k') - k' \\ &= i - 2 - d_{k'} \\ &\geq i \end{aligned}$$

Case 4: $k' > 1$ and $j - k' + 1 < i < j$. Here, $d_{k'+i-j} > d_{k'}$ and so

$$\begin{aligned} c_i^{A'_{k'}} &= c_j^A + c_{k'+i-1-j}^A - c_{k'-1}^A \\ &= j - 1 + (c_{k'+i-1-j} - k' - i + j) + k' + i - j - (c_{k'-1}^A - k') - k' \\ &= d_{k'+i-j} - d_{k'} + i - 1 \\ &\geq i \end{aligned}$$

Case 1 shows that when $k' = 1$, then $A'_1 = A$ satisfies condition 2 and is thus a valid transmission sequence. Cases 2-4 show that when $k' > 1$, $A'_{k'}$ is a valid transmission sequence because it satisfies condition 2 for the full range of $1 \leq i < j$. This proves that a valid permutation sequence exists amongst the cyclic permutations of A.

We now only need to show that for a valid transmission sequence, $B = b_1 b_2 \ldots b_j$, all cyclic permutations of B'_k of B are invalid transmission sequences. Using analogous notation to the above,

$$\begin{aligned} c_{j-k+1}^{B'_k} &= c_j^B - c_{k-1}^B \\ &= j - 1 - c_{k-1}^B \\ &\leq j - k. \end{aligned}$$

Therefore, $c_{j-k+1}^{B'_k}$ does not satisfy condition 2 and so B'_k is an invalid transmission sequence.

Given the independent and identically distributed assumption of our branching process model, the probability of an ordered set of j individuals having offspring according to sequence A is identical to the probability of the same individuals having offspring according to a rotated permutation of A. It follows from theorem 1 that the probability of having a stuttering chain of size j is $1/j$ times the probability of j individuals causing $j-1$ infections. Therefore the probability of having a stuttering chain of size j is the $(j-1)$th coefficient of $T_j(s) = \frac{1}{j}\left[Q(s)\right]^j$. Differentiation of $T_j(s)$ yields the result provided in the main text,

$$r_j = \frac{1}{(j-1)!} T_j^{(j-1)}|_{s=0}.$$
