# Supplementary material for: Inference of R 0 and Transmission Heterogeneity from the Size Distribution of Stuttering Chains
Source: PLoS Comput Biol. 2013 May 2;9(5):e1002993. doi: 10.1371/journal.pcbi.1002993 (PMC3642075; doi:10.1371/journal.pcbi.1002993)
Supplement: Text S2 — Matlab code for two key functions is provided. One function shows how we simulate the transmission and observation process models presented in this manuscript. The other function shows how the probability density can be analytically calculated for a given set of transmission parameters. This probability density can then be used to calculate the likelihood function used in all of our ML analyses. (PDF) [file pcbi.1002993.s003.pdf]

Matlab functions for:

1. Simulating the size of observed transmission chains when the offspring distribution is a negative binomial and observation of cases occurs with either an independent or chain-size dependent probability.
2. Calculating the probability density function for the observed size of simulated chains.

%%%

```
function chain_dist = chain_distribution(r0,k,pobs,max_outbreak_size,num_sim)
    % Generates disease outbreak data (i.e. the distribution for the observed
    % size of transmission chains)
    %
    % r0 = mean secondary infections per infected individual
    % k = dispersion parameter of offspring distribution
    % pobs = Observiaton bias
    %   pobs(1) = mode of bias [0 perfect surveillance, 1 i.i.d. bias
    %                              2 weighted cluster bias]
    %   pobs(2) = individual level probability of observation
    % num_sim = number of chains simulated
    %
    % chain_dist(i,1) = number of chains of size i
    % chain_dist(i,2) = number of chains of size i that did not go extinct
    %   before simulation ended

    chain_dist=zeros(1,2);

    n = 0; % Counts how many
    while (n < num_sim)
        popsize = 1; % Number of cases that can still transmit
        outbreak_size = popsize; % Running count of case in a chain
        while (popsize > 0 && outbreak_size < max_outbreak_size)
            nb_dist = gen_nb_dist(r0,k,popsize,1);
            popsize = sum(nb_dist);
            outbreak_size = outbreak_size + popsize;
        end
        if pobs(1) == 1 % i.i.d case
            outbreak_size = binornd(outbreak_size,pobs(2));
        elseif pobs(1) == 2 % weighted cluster case
            if(rand() < (1-pobs(2))^outbreak_size)
                outbreak_size = 0;
            end
        end

        % Record outbreak_size
        if (outbreak_size > 0)
            if (outbreak_size > size(chain_dist,1));
                chain_dist(outbreak_size,1) = 0;
            end
            chain_dist(outbreak_size,1) = chain_dist(outbreak_size,1)+ 1;
            if (popsize> 0)
                chain_dist(outbreak_size,2) = chain_dist(outbreak_size,2)+ 1;
            end
            n = n+1;
        end
    end
```

```
end

function nb_dist =gen_nb_dist(r0,k,m,n)
    % Chooses an m by n array of integers according to a negative binomial
    % distribution
    %
    % r0 = mean
    % k = dispersion parameter as defined in Jamie's '05 paper
    %   k =1 --> geometric dist w/ Var = ro(r0 +1)
    %   k = Inf --> Poisson dist w/ Var = r0
    %
    % nb_dist = output

    nb_dist = poissrnd(gamrnd(k,r0/k,m,n));

    % Note that matlab uses r,p for the negative binomial parameters where r
    % is 'k' and p is 'k/(r0+k)'
end

%%%%%%%%%%%%%%%%%%%%%%%%%%%%%%%%%%%%%%%%%%%%%%%%%%

function chain_pdf = calc_chain_pdf_forSup(r0,k,pobs_arr,outbreak_size_limit)
    % Calculates the expected pdf for the distribution of outbreak sizes.
    %   pobs_arr(1) = mode of bias [0 perfect surveillance, 1 i.i.d. bias
    %                                2 weighted cluster bias]
    %   pobs_arr(2) = individual level probability of observation

    pobs_mode = pobs_arr(1);
    pobs = pobs_arr(2);

    if(pobs_mode == 1 && pobs < 1)
        % Large chains can be as observed chains and so we'll have to compute
        % the 'true' pdf for chains that are larger than the maximum
        % observable size.  The choice of how large to calculate is somewhat
        % arbitrary.
        num_calc = min(100*outbreak_size_limit,1e4);
    else
        num_calc = outbreak_size_limit;
    end



    % Outline of method:
    % - Determine true pdf of chains
    % - Adjust for imperfect observation

    if r0 == 0
        true_chain_pdf = zeros(1,num_calc);
        true_chain_pdf(1) = 1;
    else
        j = 1:num_calc;
        log_real_chain_pdf = gammaln(k*j+j-1)-gammaln(k*j)-gammaln(j+1)+(j-
1)*log(r0/k)-(k*j+j-1)*log(1+r0/k);
        true_chain_pdf = exp(log_real_chain_pdf);
    end
```

```
    if (pobs_mode == 0 || pobs == 1)
        chain_pdf = true_chain_pdf;
        return;
    end

    j=1:num_calc;
    % Calculate probability a chain is not observed at all
    prob0 = sum(exp(j*log(1-pobs)+log(true_chain_pdf)));
    denominator = 1- prob0;
    switch pobs_mode
        case 1 % i.i.d. case
            for jj = 1:outbreak_size_limit
                l = jj:length(true_chain_pdf);
                numerator(jj) = exp(jj*log(pobs/(1-pobs))-
gammaln(jj+1))*sum(exp(log(true_chain_pdf(l))+l*log(1-pobs)+gammaln(l+1)-
gammaln(l+1-jj)));
            end
            chain_pdf = numerator/denominator;
        case 2 % weighted cluster
            j = 1:length(true_chain_pdf);
            numerator = exp(log(true_chain_pdf(j))).*(1-(1-pobs).^j);
            chain_pdf = numerator/denominator;
end
```
